# Trends in alcohol use and alcoholic liver disease in South Korea: a nationwide cohort study

**DOI:** 10.1186/s12889-024-19321-z

**Published:** 2024-07-10

**Authors:** Jeong-Ju Yoo, Dong Hyeon Lee, Young Chang, Hoongil Jo, Young Youn Cho, Sangheun Lee, Log Young Kim, Jae Young Jang, the Korean Association for the Study of the Liver

**Affiliations:** 1 Department of Internal Medicine, Soonchunhyang University Bucheon Hospital, Bucheon, South Korea; 2 Department of Internal Medicine, Seoul Metropolitan Government Seoul National University Boramae Medical Center, Seoul, South Korea; 3 Department of Internal Medicine, Institute for Digestive Research, Digestive Disease Center, Soonchunhyang University College of Medicine, 59 Daesagwan-Ro, Yongsan-Gu, Seoul, 04401 Republic of Korea; 4 Department of Internal Medicine, Wonkwang University School of Medicine & Hospital, Iksan, South Korea; 5 Department of Internal Medicine, Chung-Ang University Hospital, Seoul, South Korea; 6 Department of Internal Medicine, Catholic Kwandong University College of Medicine, Gangneung, South Korea; 7 Department of Big DATA Strategy, National Health Insurance Service, 32, Geongang-Ro, Wonju-si, 26464 Republic of Korea

**Keywords:** Gender, Smoking, Alcoholic liver disease, Epidemiology

## Abstract

**Background:**

There is a lack of national-level research on alcohol consumption and the epidemiology of alcoholic liver disease (ALD) in South Korea. This study aims to address the critical public health issue of ALD by focusing on its trends, incidence, and outcomes, using nationwide claims data.

**Methods:**

Utilizing National Health Insurance Service data from 2011 to 2017, we calculated the population's overall drinking amount and the incidence of ALD based on ICD-10 diagnosis codes.

**Results:**

From 2011 to 2017 in South Korea, social drinking increased from 15.7% to 16.5%, notably rising among women. High-risk drinking remained around 16.4%, decreasing in men aged 20–39 but not decreased in men aged 40–59 and steadily increased in women aged 20–59. The prevalence of ALD in high-risk drinkers (0.97%) was significantly higher than in social drinkers (0.16%). A 3-year follow-up revealed ALD incidence of 1.90% for high-risk drinkers and 0.31% for social drinkers. Women high-risk drinkers had a higher ALD risk ratio (6.08) than men (4.18). The economic burden of ALD was substantial, leading to higher healthcare costs and increased hospitalization. Progression rates to liver cirrhosis and hepatocellular carcinoma (HCC) in ALD patients were 23.3% and 2.8%, respectively, with no gender difference in cirrhosis progression.

**Conclusions:**

The study revealed a concerning rise in alcohol consumption among South Korean women and emphasizes the heightened health risks and economic burdens associated with high-risk drinking, especially concerning ALD and its complications.

**Supplementary Information:**

The online version contains supplementary material available at 10.1186/s12889-024-19321-z.

## Background

In South Korea, chronic hepatitis B and alcohol are the most common causes of liver disease in South Korea, accounting for 60–80% and 13–14.5% of cases [1–3]. Recently, there has been a decline in hepatitis B and C, while the prevalence of alcoholic liver disease (ALD) is gradually increasing [4–6]. This upward trend is concerning due to the potential for ALD to progress to severe liver conditions, including cirrhosis and hepatocellular carcinoma (HCC) [7, 8]. Alcohol consumption has a well-documented relationship with the incidence of alcoholic liver disorders. Alcoholic liver disease is a significant issue in Asia, where trends in alcohol consumption are particularly alarming. In China, the rate of alcohol consumption is increasing faster than in other regions of the world, highlighting a growing public health concern [9]. Additionally, Central Asia has recorded the highest number of alcohol-attributable liver cirrhosis Disability-Adjusted Life Years (DALYs) per 100,000 people for both men and women [10]. Research has shown that genetic factors, such as the prevalence of certain alcohol dehydrogenase and aldehyde dehydrogenase enzyme variants in Asians, contribute to a higher susceptibility to alcoholic liver diseases compared to other populations. This genetic predisposition results in a faster conversion of alcohol into acetaldehyde, a toxic metabolite, and a slower process of clearing it from the body, leading to increased liver damage from smaller amounts of alcohol.

Traditionally, alcohol has played a pivotal role in both social and business settings in South Korea. Drinking patterns in South Korea are characterized by a mix of solitary and social drinking, often involving both mixed and single-type alcohol consumption. Social drinking is commonly practiced in a variety of settings, including family gatherings, pubs, and restaurants, reflecting its integral role in both personal and professional interactions. This pattern is deeply embedded in the culture, where drinks like soju and beer are frequently consumed in combination during social occasions to facilitate bonding and business negotiations. On the other hand, solitary drinking has been on the rise, often driven by stress or social isolation, marking a shift in traditional drinking behaviors. These changes are not just social trends; they carry significant implications for public health, particularly in the incidence and progression of ALD. Additionally, the landscape of alcoholic beverage policies in South Korea has seen adjustments during the study period. These include modifications in taxation, advertising regulations, and sales restrictions aimed at curbing excessive alcohol consumption.

Unlike other viral hepatitis cases, ALD has identifiable triggering factors and is significantly influenced by social and economic policies [11]. Therefore, early identification of drinking status can aid in ALD prevention through abstinence education, effectively averting progression to liver cirrhosis or HCC [12]. Understanding the current status of drinking rates and their direct impact on ALD epidemiology is crucial for developing effective public health interventions and policies [13]. Unfortunately, no large-scale study representative of South Korea's ALD epidemiology has been conducted to date [1, 14]. This study aims to investigate drinking rates and ALD epidemiology in South Korea, examining patterns of change over time using national cohort and National Health Insurance data. Additionally, in line with existing reports that alcohol consumption varies by age and gender [15–17], we have conducted further stratified analysis based on these demographic factors

## Methods

### Data source and study population

In this study, two databases were utilized. First, to comprehend the current drinking landscape in South Korea and assess the risk levels based on drinking rates, we analyzed examination data from the National Health Insurance Corporation (National Health Insurance Service-Health Screening Cohort; NHIS-HEALs). Due to security and data capacity limitations, the complete NHIS-HEALs dataset was unavailable. Consequently, a representative sample cohort, constituting 10% of the NHIS population, was randomly selected annually from 2011 to 2017, as detailed in Supplementary Table 1. This sample cohort accurately mirrors the broader South Korean population, deliberately chosen to match the age and gender distribution of the entire NHIS-HEALs. Second, the epidemiology of patients with ALD was further validated using claims data in conjunction with NHIS-HEALs. Data reliability was ensured through two methods. Initially, the ALD incidence rate was calculated and compared from NHIS-HEALs and claims data, confirming a consistent pattern. Additionally, the proportion of high-risk drinkers was compared between National Health and Nutrition Examination Survey data and NHIS-HEALs, demonstrating consistent proportions of high-risk drinkers in the two cohorts.

Both databases contain anonymized data, including demographic details and claims information aligned with the International Classification of Diseases, 10th revision (ICD-10). The Institutional Review Board of Soonchunhyang University Bucheon Hospital approved the current study (IRB No. SCHBC 2023–05-007, approval date 23-May-2023). Informed consent was waived by the IRB since only de-identified information was utilized. Our study adhered to the ethical guidelines of the World Medical Association Declaration of Helsinki.

### Classification of alcohol drinking

In South Korea, a national health screening is conducted every two years, during which citizens are required to fill out a health questionnaire. The questionnaire includes the following items related to alcohol consumption:

1. On average, how many days per week do you drink alcohol?
2. On days when you drink, how much do you typically consume in a day? (number of drinks)

(Calculate using each type of drink's standard serving size. Note that one can of beer (355 cc) is equivalent to 1.6 standard beer servings.)

Based on these survey items, high-risk drinking was categorized as consuming alcohol more than twice weekly, with men consuming over 7 standard drinks and women more than 5, following the guidelines of the South Korean Ministry of Health and Welfare [18]. A standard drink in Korea is defined as containing 7 g of pure alcohol, in accordance with South Korean alcohol consumption guidelines [19]. For the purpose of comparison, social drinkers served as the control group for high-risk drinking. Social drinkers were identified as individuals who drink once a week, with men having up to 6 standard drinks and women up to 4 standard drinks [18].

### Outcomes

The study focused on examining the prevalence and incidence of ALD, cirrhosis, and HCC. ALD was identified in patients who received outpatient treatment more than twice or were admitted to the hospital at least once with a primary diagnosis coded under ICD-10 codes K70 (K700, K701, K702, K703, K704, and K709). Liver cirrhosis was categorized using ICD-10 codes K74, K702, and K703, while HCC was defined by the code C220. Mortality encompassed all reported deaths, regardless of the cause. Incidence referred to the emergence of a new case of the outcome during a 3-year follow-up of the sample cohort.

### Statistical analysis

Continuous variables were presented as means with standard deviations (SDs), while categorical variables were expressed as percentages, unless otherwise specified. Group differences were assessed using Student's t-test for continuous variables and the *χ*^2^ test for categorical variables. We conducted age-period-cohort (APC) analyses to identify changes in outcomes over time, accounting for the influences of age and birth cohort. Statistical analyses were performed using SAS version 9.4 (SAS Institute, Cary, NC, USA) and R version 3.2.3 (The R Foundation for Statistical Computing, Vienna, Austria, http://www.Rproject.org). A P value of less than 0.05, determined from a two-sided test, was considered indicative of statistical significance.

## Results

### Alcohol consumption trends in South Korea

The proportion of social drinkers was 15.7% in 2011, gradually increasing thereafter and reaching 16.5% in 2017 (*p* for trend < 0.001) (Table 1, Supplementary Fig. 1). For men, the proportion of social drinkers peaked at 16.8% in 2014 and has been decreasing since, while for women, it has shown a consistent upward trend each year. Meanwhile, high-risk drinkers remained constant at about 16.5% from 2011 to 2017. Although the number of high-risk drinkers among men gradually decreased, the proportions among women increased annually. When analyzed by age and gender, the proportion of high-risk drinkers decreased among men aged 20–39, while it does not decrease among men aged 40–59. In women, the number of high-risk drinkers increased each year across all age groups from 20 to 59. To validate the reliability of NHIS-HEALs data, the proportion of high-risk drinkers was cross-verified with National Health and Nutrition Examination Survey data, revealing consistent proportions in both cohorts (Supplementary Table 2).

**Table 1:** Drinking rate

		2011		2012		2013		2014		2015		2016		2017	
Sex	Age	Proportion (%)	SE (%)	Proportion (%)	SE (%)	Proportion (%)	SE (%)	Proportion (%)	SE (%)	Proportion (%)	SE (%)	Proportion (%)	SE (%)	Proportion (%)	SE (%)
**Social drinker**															
Male	20-39	20.1	0.02	20.8	0.02	21.3	0.03	21.7	0.03	21.0	0.03	20.6	0.03	20.5	0.03
	40-59	15.3	0.02	15.9	0.02	15.8	0.02	16.5	0.02	16.1	0.02	16.0	0.02	16.2	0.02
	≥ 60	11.3	0.04	11.8	0.04	11.7	0.04	12.1	0.04	12.4	0.04	12.5	0.04	12.8	0.03
	SUM	15.8	0.01	16.3	0.01	16.4	0.01	16.8	0.01	16.6	0.01	16.4	0.01	16.6	0.01
Female	20-39	20.8	0.03	21.3	0.03	21.7	0.03	22.6	0.03	22.3	0.03	22.3	0.03	22.8	0.03
	40-59	13.1	0.02	13.3	0.02	13.7	0.02	14.4	0.02	14.8	0.02	15.0	0.02	15.6	0.02
	≥ 60	4.0	0.04	4.1	0.04	4.0	0.03	4.2	0.03	4.4	0.03	4.6	0.03	4.9	0.03
	SUM	15.3	0.01	15.7	0.01	15.7	0.01	16.3	0.01	16.1	0.01	15.9	0.01	16.2	0.01
Total		15.7	0.01	16.1	0.01	16.2	0.01	16.7	0.01	16.4	0.01	16.3	0.01	16.5	0.01
**High-risk drinker**															
Male	20-39	25.7	0.02	24.9	0.02	24.4	0.03	24.1	0.03	24.4	0.03	23.8	0.03	23.6	0.03
	40-59	27.3	0.02	27.0	0.02	27.0	0.02	26.9	0.02	27.5	0.02	27.4	0.02	27.3	0.02
	≥ 60	14.3	0.04	14.1	0.04	13.9	0.04	14.0	0.04	13.9	0.04	14.0	0.04	14.1	0.03
	SUM	18.1	0.01	17.7	0.01	17.6	0.01	17.5	0.01	17.8	0.01	17.6	0.01	17.6	0.01
Female	20-39	8.9	0.03	8.7	0.03	9.4	0.03	9.4	0.03	10.4	0.03	10.5	0.03	11.0	0.03
	40-59	4.1	0.02	4.2	0.02	4.3	0.02	4.4	0.02	4.8	0.02	4.9	0.02	5.1	0.02
	≥ 60	0.7	0.04	0.8	0.04	0.8	0.03	0.9	0.03	0.9	0.03	1.0	0.03	1.1	0.03
	SUM	16.0	0.01	15.7	0.01	15.7	0.01	15.7	0.01	16.0	0.01	15.9	0.01	15.9	0.01
Total		16.8	0.01	16.5	0.01	16.4	0.01	16.3	0.01	16.7	0.01	16.5	0.01	16.4	0.01

### Consequences of alcoholic liver disease caused by alcohol consumption

Subsequently, we assessed the occurrence of ALD, liver cirrhosis, HCC, and mortality based on the volume of alcohol consumed. The prevalence of ALD among high-risk drinkers was 0.97%, significantly surpassing the prevalence of 0.16% among social drinkers (Supplementary Table 3). Over a 3-year follow-up, the incidence of ALD in high-risk drinkers reached 1.90%, while in social drinkers, it was 0.31% (Table 2). In both groups, ALD incidence rose with age and was higher in men than in women. The prevalence of cirrhosis was 0.19% among high-risk drinkers, exceeding the 0.10% among social drinkers (Supplementary Table 4). The 3-year follow-up also revealed a higher incidence of cirrhosis in high-risk drinkers (0.43% vs. 0.19%) (Table 2). The patterns for the prevalence (Supplementary Table 5) and incidence (Table 4) of HCC followed a similar trend, with significantly higher rates in high-risk drinkers compared to social drinkers (prevalence: 0.04% vs. 0.03%, incidence: 0.13% vs. 0.08%). Furthermore, 3-year mortality was elevated in high-risk drinkers (0.50% vs. 0.24%) (Supplementary Table 6).

**Table 2:** Consequences of alcoholic liver disease caused by alcohol consumption over 3 years

		2011 3-year follow-up (2012–2014)			2012 3-year follow-up (2013–2015)			2013 3-year follow-up (2014–2016)			2014 3-year follow-up (2015–2017)		
Sex	Age	Cohort	Incidence (n)	Incidence rate (%)	Cohort	Incidence (n)	Incidence rate (%)	Cohort	Incidence (n)	Incidence rate (%)	Cohort	Incidence (n)	Incidence rate (%)
**Incidence of alcoholic liver disease over 3 years**													
**Social drinker**													
Male	20—39	154,589	588	0.38	158,398	488	0.31	160,032	506	0.32	161,487	457	0.28
	40—59	122,963	1087	0.88	130,725	1086	0.83	131,720	1029	0.78	139,775	1026	0.73
	≥ 60	24,414	272	1.11	26,051	295	1.13	26,615	278	1.04	28,979	302	1.04
	SUM	301,966	1947	0.64	315,174	1869	0.59	318,367	1813	0.57	330,241	1785	0.54
Female	20—39	151,040	126	0.08	152,888	106	0.07	153,328	107	0.07	157,632	107	0.07
	40—59	103,148	257	0.25	107,058	275	0.26	112,423	238	0.21	120,091	215	0.18
	≥ 60	10,475	42	0.40	10,931	44	0.40	11,346	29	0.26	12,550	42	0.33
	SUM	264,663	425	0.16	270,877	425	0.16	277,097	374	0.13	290,273	364	0.13
Total		566,629	2372	0.42	586,051	2294	0.39	595,464	2187	0.37	620,514	2149	0.35
**High risk drinker**													
Male	20—39	197,895	2307	1.17	189,676	1969	1.04	183,502	2014	1.10	179,741	1935	1.08
	40—59	219,140	7070	3.23	221,692	6947	3.13	225,866	6906	3.06	228,531	6732	2.95
	≥ 60	32,768	1638	5.00	33,471	1640	4.90	34,261	1703	4.97	36,497	1693	4.64
	SUM	449,803	11,015	2.45	444,839	10,556	2.37	443,629	10,623	2.39	444,769	10,360	2.33
Female	20—39	64,541	234	0.36	62,455	208	0.33	66,229	227	0.34	65,701	229	0.35
	40—59	32,555	521	1.60	33,585	580	1.73	35,566	573	1.61	36,912	605	1.64
	≥ 60	2133	39	1.83	2509	44	1.75	2621	56	2.14	3023	62	2.05
	SUM	99,229	794	0.80	98,549	832	0.84	104,416	856	0.82	105,636	896	0.85
Total		549,032	11,809	2.15	543,388	11,388	2.10	548,045	11,479	2.09	550,405	11,256	2.05
**Incidence of liver cirrhosis over 3 years**													
**Social drinker**													
Male	20—39	154,589	88	0.06	158,398	96	0.06	160,032	90	0.06	161,487	95	0.06
	40—59	122,963	561	0.46	130,725	574	0.44	131,720	598	0.45	139,775	641	0.46
	≥ 60	24,414	161	0.66	26,051	177	0.68	26,615	197	0.74	28,979	204	0.70
	SUM	301,966	810	0.27	315,174	847	0.27	318,367	885	0.28	330,241	940	0.28
Female	20—39	151,040	33	0.02	152,888	29	0.02	153,328	42	0.03	157,632	21	0.01
	40—59	103,148	147	0.14	107,058	141	0.13	112,423	150	0.13	120,091	173	0.14
	≥ 60	10,475	35	0.33	10,931	34	0.31	11,346	41	0.36	12,550	42	0.33
	SUM	264,663	215	0.08	270,877	204	0.08	277,097	233	0.08	290,273	236	0.08
Total		566,629	1025	0.18	586,051	1051	0.18	595,464	1118	0.19	620,514	1176	0.19
**High risk drinker**													
Male	20—39	197,895	120	0.06	189,676	104	0.05	183,502	125	0.07	179,741	126	0.07
	40—59	219,140	1417	0.65	221,692	1400	0.63	225,866	1525	0.68	228,531	1478	0.65
	≥ 60	32,768	493	1.50	33,471	511	1.53	34,261	516	1.51	36,497	483	1.32
	SUM	449,803	2030	0.45	444,839	2015	0.45	443,629	2166	0.49	444,769	2087	0.47
Female	20—39	64,541	24	0.04	62,455	31	0.05	66,229	27	0.04	65,701	31	0.05
	40—59	32,555	108	0.33	33,585	134	0.40	35,566	133	0.37	36,912	165	0.45
	≥ 60	2133	17	0.80	2509	19	0.76	2621	22	0.84	3023	22	0.73
	SUM	99,229	149	0.15	98,549	184	0.19	104,416	182	0.17	105,636	218	0.21
Total		549,032	2179	0.40	543,388	2199	0.40	548,045	2348	0.43	550,405	2305	0.42
**Incidence of hepatocellular carcinoma over 3 years**													
**Social drinker**													
Male	20—39	154,589	30	0.02	158,398	23	0.01	160,032	24	0.01	161,487	22	0.01
	40—59	122,963	210	0.17	130,725	278	0.21	131,720	232	0.18	139,775	272	0.19
	≥ 60	24,414	99	0.41	26,051	112	0.43	26,615	120	0.45	28,979	127	0.44
	SUM	301,966	339	0.11	315,174	413	0.13	318,367	376	0.12	330,241	421	0.13
Female	20—39	151,040	8	0.01	152,888	5	0.00	153,328	9	0.01	157,632	6	0.00
	40—59	103,148	48	0.05	107,058	29	0.03	112,423	35	0.03	120,091	38	0.03
	≥ 60	10,475	14	0.13	10,931	11	0.10	11,346	10	0.09	12,550	13	0.10
	SUM	264,663	70	0.03	270,877	45	0.02	277,097	54	0.02	290,273	57	0.02
Total		566,629	409	0.07	586,051	458	0.08	595,464	430	0.07	620,514	478	0.08
**High risk drinker**													
Male	20—39	197,895	26	0.01	189,676	24	0.01	183,502	35	0.02	179,741	35	0.02
	40—59	219,140	471	0.21	221,692	434	0.20	225,866	460	0.20	228,531	458	0.20
	≥ 60	32,768	213	0.65	33,471	187	0.56	34,261	193	0.56	36,497	203	0.56
	SUM	449,803	710	0.16	444,839	645	0.14	443,629	688	0.16	444,769	696	0.16
Female	20—39	64,541	8	0.01	62,455	6	0.01	66,229	2	0.00	65,701	2	0.00
	40—59	32,555	20	0.06	33,585	23	0.07	35,566	22	0.06	36,912	27	0.07
	≥ 60	2133	2	0.09	2509	7	0.28	2621	1	0.04	3023	9	0.30
	SUM	99,229	30	0.03	98,549	36	0.04	104,416	25	0.02	105,636	38	0.04
Total		549,032	740	0.13	543,388	681	0.13	548,045	713	0.13	550,405	734	0.13

		2015 3-year follow-up (2016–2018)			2016 3-year follow-up (2017–2019)			2017 3-year follow-up (2018–2020)		
Sex	Age	Cohort	Incidence (n)	Incidence rate (%)	Cohort	Incidence (n)	Incidence rate (%)	Cohort	Incidence (n)	Incidence rate (%)
**Incidence of alcoholic liver disease over 3 years**										
**Social drinker**										
Male	20—39	155,424	475	0.31	152,067	411	0.27	150,728	343	0.23
	40—59	137,774	980	0.71	137,377	880	0.64	139,195	865	0.62
	≥ 60	31,635	261	0.83	33,711	345	1.02	36,530	343	0.94
	SUM	324,833	1716	0.53	323,155	1636	0.51	326,453	1551	0.48
Female	20—39	154,143	77	0.05	152,646	82	0.05	155,215	80	0.05
	40—59	123,611	220	0.18	125,555	235	0.19	131,359	249	0.19
	≥ 60	14,074	34	0.24	15,498	58	0.37	17,465	64	0.37
	SUM	291,828	331	0.11	293,699	375	0.13	304,039	393	0.13
Total		616,661	2047	0.33	616,854	2011	0.33	630,492	1944	0.31
**High risk drinker**										
Male	20—39	180,484	1828	1.01	175,245	1811	1.03	173,058	1708	0.99
	40—59	235,420	6916	2.94	234,602	6637	2.83	234,079	6310	2.70
	≥ 60	39,022	1797	4.61	41,835	1868	4.47	44,853	1901	4.24
	SUM	454,926	10,541	2.32	451,682	10,316	2.28	451,990	9919	2.19
Female	20—39	71,949	269	0.37	71,875	277	0.39	75,082	271	0.36
	40—59	40,035	592	1.48	41,261	639	1.55	42,455	652	1.54
	≥ 60	3401	60	1.76	3904	80	2.05	4351	76	1.75
	SUM	115,385	921	0.80	117,040	996	0.85	121,888	999	0.82
Total		570,311	11,462	2.01	568,722	11,312	1.99	573,878	10,918	1.90
**Incidence of liver cirrhosis over 3 years**										
**Social drinker**										
Male	20—39	155,424	87	0.06	152,067	93	0.06	150,728	85	0.06
	40—59	137,774	615	0.45	137,377	599	0.44	139,195	537	0.39
	≥ 60	31,635	201	0.64	33,711	241	0.71	36,530	272	0.74
	SUM	324,833	903	0.28	323,155	933	0.29	326,453	894	0.27
Female	20—39	154,143	23	0.01	152,646	30	0.02	155,215	24	0.02
	40—59	123,611	154	0.12	125,555	166	0.13	131,359	182	0.14
	≥ 60	14,074	41	0.29	15,498	55	0.35	17,465	73	0.42
	SUM	291,828	218	0.07	293,699	251	0.09	304,039	279	0.09
Total		616,661	1121	0.18	616,854	1184	0.19	630,492	1173	0.19
**High risk drinker**										
Male	20—39	180,484	121	0.07	175,245	119	0.07	173,058	95	0.05
	40—59	235,420	1558	0.66	234,602	1480	0.63	234,079	1477	0.63
	≥ 60	39,022	574	1.47	41,835	687	1.64	44,853	664	1.48
	SUM	454,926	2253	0.50	451,682	2286	0.51	451,990	2236	0.49
Female	20—39	71,949	30	0.04	71,875	39	0.05	75,082	34	0.05
	40—59	40,035	160	0.40	41,261	190	0.46	42,455	191	0.45
	≥ 60	3401	26	0.76	3904	37	0.95	4351	31	0.71
	SUM	115,385	216	0.19	117,040	266	0.23	121,888	256	0.21
Total		570,311	2469	0.43	568,722	2552	0.45	573,878	2492	0.43
**Incidence of hepatocellular carcinoma over 3 years**										
**Social drinker**										
Male	20—39	155,424	15	0.01	152,067	20	0.01	150,728	17	0.01
	40—59	137,774	275	0.20	137,377	257	0.19	139,195	234	0.17
	≥ 60	31,635	134	0.42	33,711	151	0.45	36,530	161	0.44
	SUM	324,833	424	0.13	323,155	428	0.13	326,453	412	0.13
Female	20—39	154,143	8	0.01	152,646	8	0.01	155,215	5	0.00
	40—59	123,611	46	0.04	125,555	58	0.05	131,359	46	0.04
	≥ 60	14,074	12	0.09	15,498	16	0.10	17,465	17	0.10
	SUM	291,828	66	0.02	293,699	82	0.03	304,039	68	0.02
Total		616,661	490	0.08	616,854	510	0.08	630,492	480	0.08
**High risk drinker**										
Male	20—39	180,484	33	0.02	175,245	36	0.02	173,058	21	0.01
	40—59	235,420	473	0.20	234,602	432	0.18	234,079	421	0.18
	≥ 60	39,022	226	0.58	41,835	255	0.61	44,853	234	0.52
	SUM	454,926	732	0.16	451,682	723	0.16	451,990	676	0.15
Female	20—39	71,949	2	0.00	71,875	3	0.00	75,082	4	0.01
	40—59	40,035	30	0.07	41,261	24	0.06	42,455	29	0.07
	≥ 60	3401	4	0.12	3904	8	0.20	4351	12	0.28
	SUM	115,385	36	0.03	117,040	35	0.03	121,888	45	0.04
Total		570,311	768	0.13	568,722	758	0.13	573,878	721	0.13

### Age–period–cohort analysis

**Fig. 1:**
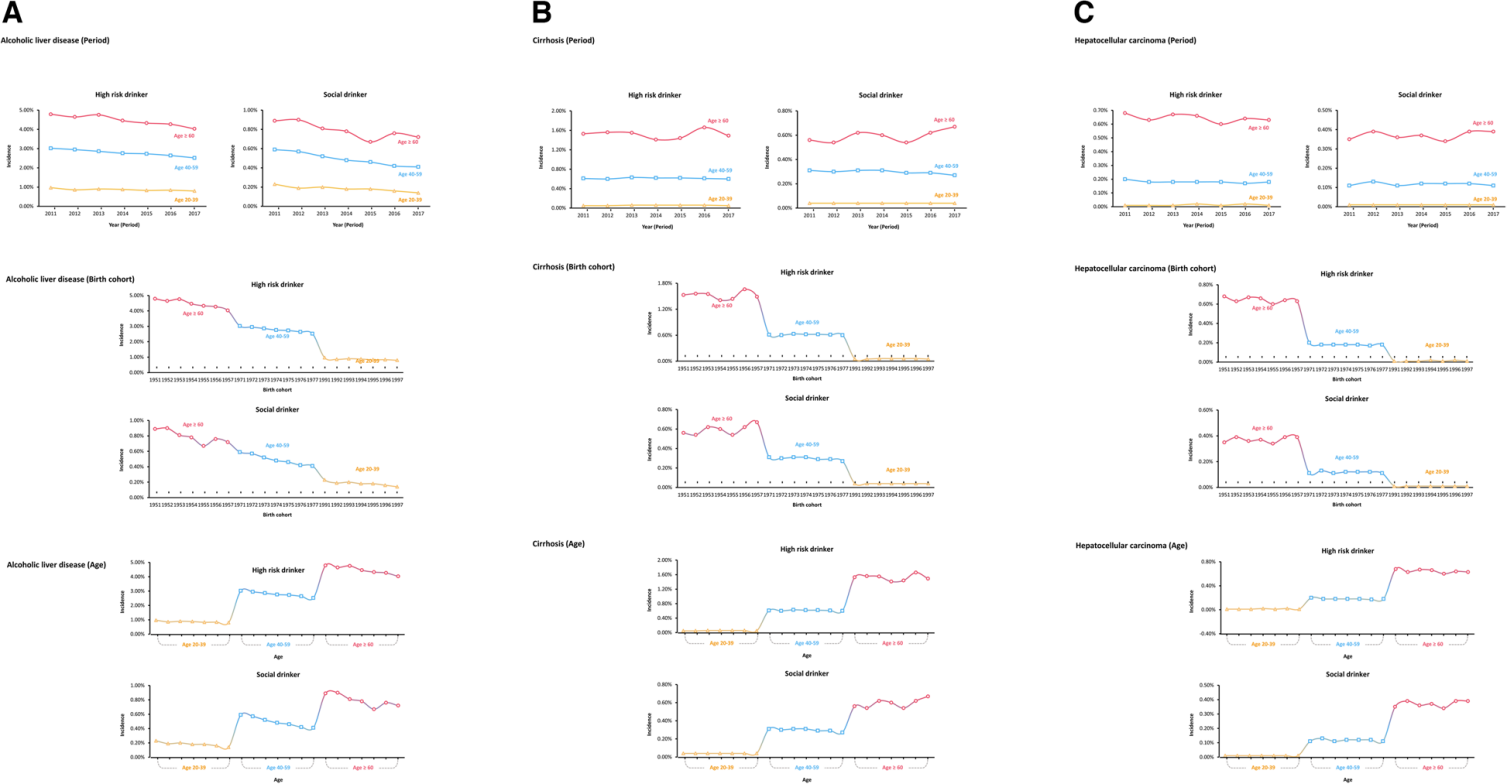
Age-period-cohort (APC) analyses. **a** alcoholic liver disease, (**b**) liver cirrhosis, (**c**) hepatocellular carcinoma

Our APC analysis of ALD, liver cirrhosis, and HCC over a three-year span reveals distinct trends among different age groups and drinking behaviors (Fig. 1). For high-risk drinkers, the incidence of ALD decreases with age, with the highest rates in older age groups. Social drinkers show consistently lower incidence rates across all conditions compared to high-risk drinkers. The incidence of liver cirrhosis and HCC is higher in older age groups for both high-risk and social drinkers, with a more pronounced increase among high-risk drinkers. Overall, while high-risk drinkers exhibit a gradual decline in incidence rates over time, social drinkers maintain relatively stable and lower rates across all age groups and conditions.

### Vulnerability of females to alcoholic liver disease

Stratified by gender, we computed the risk ratios (RRs) for ALD, cirrhosis, and HCC in high-risk drinkers compared to social drinkers. Compared to social drinkers, the risk of developing ALD was higher in women (RR 6.08) than in men (RR 4.18) (Supplementary Fig. 2A). Similarly, the risk of developing liver cirrhosis (women 2.31, men 1.74; Supplementary Fig. 2B) and HCC (women 1.48, men 1.25; Supplementary Fig. 2C) was determined to have a higher RR value in women than in men.

### Epidemiology of alcohol-associated liver disease and economic burden

**Fig. 2:**
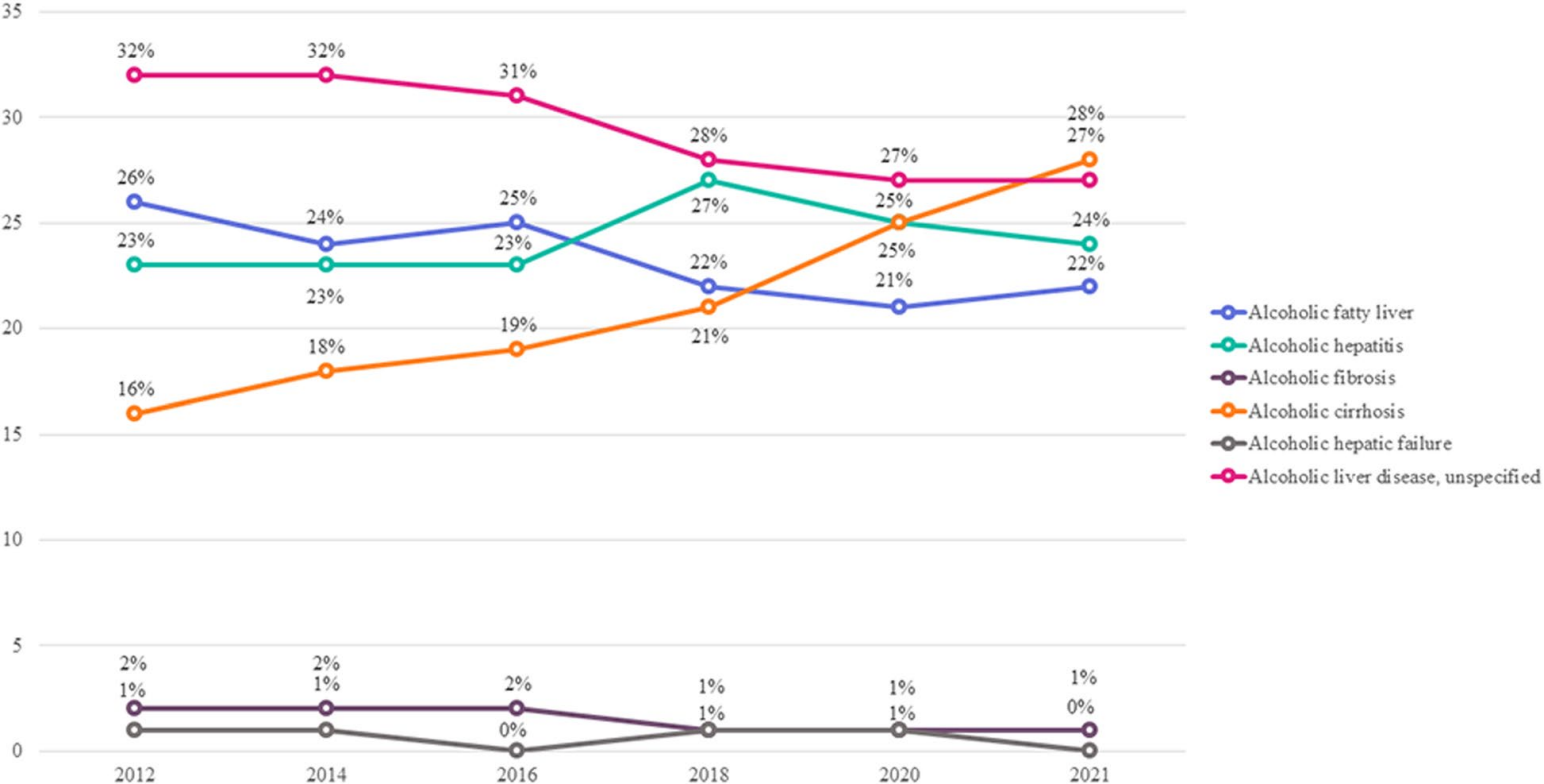
Distribution of stages in alcohol-related liver disease

Subsequently, we computed epidemiological data and economic costs associated with ALD using claims data. The incidence of ALD showed a yearly decline, decreasing from 0.39% in 2012 to 0.33% in 2017, with a more significant decrease observed in men compared to women (Supplementary Table 7). To validate the reliability of the claim data, we compared the NHIS-HEALs cohort with ALD incidence and confirmed that the trends in the two cohorts were consistent. When ALD was categorized into detailed disease codes, the proportion of relatively mild diseases such as alcoholic fatty liver, ALD, and unspecified decreased, while the proportion of liver cirrhosis increased from 16 to 27% (Fig. 2). Comparing the healthcare utilization of ALD patients with the control group, the ALD group exhibited significantly higher total medical costs and drug costs than the control group (Table 3). Additionally, the number of outpatient visits and hospitalization days in the ALD group exceeded those of the control group (Table 3, Supplementary Fig. 3A/3B). This discrepancy appears to be linked to the higher comorbidity rate in the ALD group compared to the control group (Supplementary Table 8).

**Table 3:** Annual number of patients and utilization of medical institutions in alcoholic liver disease and control groups

	2012		P	2014		P	2016		P
	ALD	Control		ALD	Control		ALD	Control	
	(*N* = 160,727)	(*N* = 642,908)		(*N* = 144,229)	(*N* = 576,916)		(*N* = 143,205)	(*N* = 572,820)	
Male (number, %)	137,618 (85.6)	550,472 (85.6)	0.999	124,590 (86.3)	498,360 (86.3)	0.999	122,950 (85.9)	491,800 (85.9)	0.999
Age	52.7 ± 11.9	52.70 ± 11.9	0.999	53.6 ± 11.8	53.65 ± 11.8	0.999	54.5 ± 11.9	54.54 ± 11.9	0.999
Annual medical cost (10^3^ KRW)	2132 ± 3715	786 ± 2229	< 0.001	2473 ± 4361	853 ± 2382	< 0.001	2820 ± 5194	983 ± 2760	< 0.001
Annual medical cost related with medication (10^3^ KRW)	684 ± 986	352 ± 781	< 0.001	721 ± 1087	363 ± 792	< 0.001	823 ± 1374	416 ± 950	< 0.001
Visit of outpatient clinic (day)	26 ± 26.1	16 ± 22	< 0.001	26 ± 27	16 ± 22	< 0.001	26 ± 26	17 ± 22	< 0.001
Hospital admission (day)	10 ± 23	2 ± 8	< 0.001	11 ± 25	2 ± 8	< 0.001	10 ± 24	2 ± 8	< 0.001

	2018		P	2020		P	2021		P
	ALD	Control		ALD	Control		ALD	Control	
	(*N* = 139,829)	(*N* = 559,316)		(*N* = 124,403)	(*N* = 497,612)		(*N* = 119,630)	(*N* = 478,520)	
Male (number, %)	118,867 (85.0)	475,468 (85.0)	0.999	104,316 (83.9)	417,264 (83.9)	0.999	99,966 (83.6)	399,864 (83.6)	0.999
Age	55.3 ± 11.9	55.3 ± 11.9	0.999	56.3 ± 12.0	56.3 ± 12.0	0.999	56.7 ± 12.0	56.7 ± 12.0	0.999
Annual medical cost (10^3^ KRW)	3447 ± 6786	1170 ± 3193	< 0.001	4076 ± 8234	1327 ± 3637	< 0.001	4351 ± 892	1477 ± 3947	< 0.001
Annual medical cost related with medication (10^3^ KRW)	915 ± 1530	467 ± 998	< 0.001	1018 ± 1703	517 ± 1093	< 0.001	1073 ± 1800	552 ± 1151	< 0.001
Visit of outpatient clinic (day)	26 ± 26	17 ± 22	< 0.001	25 ± 26	16 ± 22	< 0.001	25 ± 26	16 ± 22	< 0.001
Hospital admission (day)	9 ± 22	2 ± 7	< 0.001	9 ± 21	1 ± 7	< 0.001	8 ± 21	2 ± 7	< 0.001

### Natural history of alcohol-associated liver disease

Finally, we computed the rate of progression to liver cirrhosis or HCC in individuals diagnosed with ALD (Table 4). Over a 3-year follow-up period, the progression rates for liver cirrhosis and HCC in individuals with ALD were 23.3% and 2.8%, respectively. Notably, there was no significant difference in the rate of progression from ALD to liver cirrhosis between men and women (men 21.7%, women 21.7%; *p* = 0.382).

**Table 4:** Progression from alcoholic liver disease to cirrhosis or hepatocellular carcinoma

		2012 3-year follow-up (2013–2015)				2014 3-year follow-up (2015–2017)				2016 3-year follow-up (2017–2019)				2018 3-year follow-up (2019–2021)				2019 3-year follow-up (2020–2022)			
Sex	Age	Cohort	Incidence (*n*)	Incidence rate (%)		Cohort	Incidence (*n*)	Incidence rate (%)		Cohort	Incidence (*n*)	Incidence rate (%)		Cohort	Incidence *(n*)	Incidence rate (%)		Cohort	Incidence (*n*)	Incidence rate (%)	
**Progression to liver cirrhosis**																					
Male	20-39	18,512	809	4.37%		14,618	705	4.82%		13,325	701	5.26%		12,142	756	6.23%		11,350	702	6.19%	
	40-59	81,286	15,232	18.74%		72,744	14,849	20.41%		68,311	14,559	21.31%		62,343	13,467	21.60%		60,109	12,992	21.61%	
	60-79	36,546	8,777	24.02%		35,840	9,408	26.25%		39,624	11,118	28.06%		42,412	12,352	29.12%		44,188	12,768	28.89%	
	≥ 80	1,274	233	18.29%		1,388	325	23.41%		1,690	417	24.67%		1,970	542	27.51%		2,283	652	28.56%	
	SUM	137,618	25,051	18.20%		124,590	25,287	20.30%		122,950	26,795	21.79%		118,867	27,117	22.81%		117,930	27,114	22.99%	
Female	20-39	5,349	541	10.11%		4,033	563	13.96%		3,789	662	17.47%		3,709	721	19.44%		3,629	736	20.28%	
	40-59	12,534	2,047	16.33%		11,399	2,442	21.42%		11,768	2,858	24.29%		11,911	3,157	26.50%		12,144	3,257	26.82%	
	60-79	4,828	746	15.45%		3,865	840	21.73%		4,300	1,022	23.77%		4,956	1,210	24.41%		5,294	1,323	24.99%	
	≥ 80	398	43	10.80%		342	64	18.71%		398	69	17.34%		386	93	24.09%		433	93	21.48%	
	SUM	23,109	3,377	14.61%		19,639	3,909	19.90%		20,255	4,611	22.76%		20,962	5,181	24.72%		21,500	5,409	25.16%	
Total		160,727	28,428	17.69%		144,229	29,196	20.24%		143,205	31,406	21.93%		139,829	32,298	23.10%		139,430	32,523	23.33%	
**Progression to hepatocellular carcinoma**																					
Male	20-39	18,512	53	0.29%		14,618	32	0.22%		13,325	39	0.29%		12,142	32	0.26%		11,350	32	0.28%	
	40-59	81,286	1,612	1.98%		72,744	1,485	2.04%		68,311	1,432	2.10%		62,343	1,233	1.98%		60,109	1,233	2.05%	
	60-79	36,546	1,740	4.76%		35,840	1,786	4.98%		39,624	1,962	4.95%		42,412	2,157	5.09%		44,188	2,175	4.92%	
	≥ 80	1,274	60	4.71%		1,388	75	5.40%		1,690	117	6.92%		1,970	139	7.06%		2,283	138	6.04%	
	SUM	137,618	3,465	2.52%		124,590	3,378	2.71%		122,950	3,550	2.89%		118,867	3,561	3.00%		117,930	3,578	3.03%	
Female	20-39	5,349	41	0.77%		4,033	37	0.92%		3,789	48	1.27%		3,709	48	1.29%		3,629	46	1.27%	
	40-59	12,534	139	1.11%		11,399	163	1.43%		11,768	189	1.61%		11,911	227	1.91%		12,144	228	1.88%	
	60-79	4,828	87	1.80%		3,865	80	2.07%		4,300	96	2.23%		4,956	119	2.40%		5,294	117	2.21%	
	≥ 80	398	10	2.51%		342	7	2.05%		398	12	3.02%		386	9	2.33%		433	11	2.54%	
	SUM	23,109	277	1.20%		19,639	287	1.46%		20,255	345	1.70%		20,962	403	1.92%		21,500	402	1.87%	
Total		160,727	3,742	2.33%		144,229	3,665	2.54%		143,205	3,895	2.72%		139,829	3,964	2.83%		139,430	3,980	2.85%	

## Discussion

Our study’s key findings include the gradual increase in social drinking, rising rates of high-risk drinking in women, gender and age-specific variations in alcohol consumption patterns, and the concerning association of high-risk drinking with the prevalence and incidence of ALD, liver cirrhosis, HCC, and mortality.

The primary finding of this study is the gradual increase in the proportion of social drinkers in South Korea. To ensure the reliability of this observation, alternative definitions, such as those who drink once a week, were explored, revealing a consistent pattern (Supplementary Table 9). We posit that two sociological factors in South Korea contribute to this trend. First, the absence of stringent regulations on alcohol advertising or broadcasting allows for the widespread portrayal of drinking scenes in public broadcasts and on platforms like YouTube, fostering a relaxed and favorable attitude towards drinking [20]. Also, the increase in alcohol consumption is influenced by the extensive reach of media and advertising. Alcohol brands frequently employ popular celebrities and K-pop idols in their marketing strategies, which are prominently displayed across diverse media platforms, including television and social media. Such advertisements portray alcohol consumption as an appealing aspect of a glamorous lifestyle, which resonates strongly with young audiences. Second, the increasing popularity of low-alcohol beverages, particularly among younger demographics, compounds the issue [21].

In terms of regulatory efforts, South Korea has established policies such as imposing taxes on alcoholic beverages and regulating sales times to control alcohol consumption. However, the enforcement of these policies is often lax, and specific regulations aimed at curbing alcohol advertising are insufficiently rigorous. This creates a regulatory environment where alcohol is both easily accessible and affordably priced, further encouraging its consumption among the youth. We suggest that the existing policies need to be strengthened with stricter advertising restrictions and more consistent enforcement of alcohol sales regulations.

The secondary finding is an increase in the number of high-risk drinkers among women. The overall rise of high-risk drinking among women is not exclusive to South Korea but represents a global phenomenon [22–24]. These changes might be influenced by evolving sociocultural dynamics, such as more women participating in traditionally male-dominated professional environments, possibly adopting associated social drinking habits. Moreover, marketing strategies targeted at women by alcohol companies also play a significant role. These campaigns often promote alcoholic beverages as symbols of modernity and independence, appealing particularly to a younger, female audience. Additionally, the increasing stress levels due to rapid socio-economic changes in the country could differentially influence drinking behaviors between genders. Women might use alcohol as a coping mechanism differently than men, which warrants further exploration [25, 26].

Our APC analysis indicate that the incidence of ALD, liver cirrhosis, and HCC varies significantly not only with gender, but also with age. Younger individuals, particularly those aged 20–39, show lower incidence rates of these conditions compared to older age groups. This can be attributed to the cumulative effects of long-term alcohol consumption, which typically manifests in more severe liver conditions over time. As people age, the prolonged exposure to alcohol and its hepatotoxic effects increase the likelihood of developing ALD and its complications.

Another important finding of our study is the notable increase in the proportion of liver cirrhosis cases within the spectrum of ALD. Upon manifestation of ALD, our study revealed a 23.3% probability of progressing to liver cirrhosis within 3 years, a figure consistent across both men and women and comparable to findings in other countries [27]. Alcoholic liver cirrhosis stands as a significant global public health concern, with an estimated 25% of cirrhosis-related deaths worldwide attributed to alcohol in 2019 [24, 28]. Recently observed shifts in South Korea, where the etiology of chronic liver disease is transitioning from viral hepatitis to ALD, emphasize the imperative for sustained attention and more effective treatments for alcoholic liver cirrhosis [8, 29, 30].

Additionally, our study demonstrated the vulnerability of females to ALD. The higher RR of developing ALD, cirrhosis, and HCC in women compared to men among high-risk drinkers aligns with findings in other studies [31]. We posit that the heightened vulnerability of women to alcohol stems from a combination of biological and physiological factors. Women, on average, have a higher body fat percentage and less body water, resulting in a more concentrated presence of alcohol in their bloodstream after consuming similar amounts as men. This prolonged exposure contributes to more significant liver damage over time [32, 33]. Additionally, women exhibit lower levels of alcohol dehydrogenase, the enzyme responsible for metabolizing alcohol, resulting in an extended duration of alcohol presence in their system, exposing the liver to harmful metabolites for prolonged periods [34]. Hormonal differences, particularly involving estrogen, may enhance women's susceptibility to alcohol-induced liver injury [34, 35]. Lastly, nutritional variances and social factors also contribute to women's heightened vulnerability to ALD. Social stigma and other barriers may lead women to delay seeking treatment, resulting in more advanced liver disease at the time of diagnosis [36, 37].

In the case of ALD, psychiatric alcohol abstinence treatment is essential. In South Korea, only about 9% of ALD patients, regardless of gender, receive formal psychiatric treatment, and this percentage is further decreasing each year (Supplementary Fig. 4). The information regarding population coverage, treatment coverage (e.g., alcohol use disorder and treatment), and copayment of these patients is listed in Supplementary Table 10. Lastly, the differences in healthcare utilization between ALD and HCC are notable. Patients with ALD generally incur lower healthcare costs and have fewer hospital admissions compared to those with HCC. This is likely because HCC, being a more advanced and severe condition, requires more intensive treatments, frequent monitoring, and complex interventions such as surgery, chemotherapy, or liver transplantation. In contrast, ALD management often involves lifestyle modifications, medication, and less frequent hospital visits unless it progresses to more severe stages like cirrhosis or HCC.

Our study has several limitations. Firstly, it relies on self-reported data for alcohol consumption, which may be subject to recall bias and underreporting. Secondly, the use of ICD-10 codes for diagnosing ALD and HCC might not capture all cases accurately, as some patients may be misclassified or undiagnosed. Thirdly, the study's observational design cannot establish causality between alcohol consumption and liver disease outcomes. Additionally, the cohort is based on South Korean individuals, which may limit the generalizability of the findings to other populations with different drinking habits and genetic predispositions. Lastly, the data on alcohol consumption patterns and healthcare utilization may not fully reflect recent trends, as the study period ends in 2017, potentially overlooking changes in drinking behaviors and policy impacts in subsequent years.

In conclusion, our study assesses the dynamic trends in alcohol consumption in South Korea and their concerning association with ALD and related liver diseases. The gender-specific findings, particularly the heightened vulnerability of women to the adverse effects of high-risk drinking, warrant urgent public health strategies and policies tailored to these trends.

### Supplementary Information

Supplementary Material 1.

## Data Availability

The datasets generated during and/or analysed during the current study are available from the corresponding author on reasonable request.

## Abbreviations

ALD: Alcoholic liver disease
HCC: Hepatocellular carcinoma
ICD-10: International Classification of Diseases, 10th revision
NHIS-HEALs: National Health Insurance Service-Health Screening Cohort
RRs: Risk ratios
SDs: Standard deviations
