# Supplementary material for: Trends in alcohol use and alcoholic liver disease in South Korea: a nationwide cohort study
Source: BMC Public Health. 2024 Jul 10;24:1841. doi: 10.1186/s12889-024-19321-z (PMC11234741; doi:10.1186/s12889-024-19321-z)

**Supplementary Table 1.** National Health Insurance Service-Health Screening Cohort (NHIS-HEALs) and 10% sample cohort

|  |  | 2011 | | 2012 | | 2013 | | 2014 | | 2015 | | 2016 | | 2017 | |
| --- | --- | --- | --- | --- | --- | --- | --- | --- | --- | --- | --- | --- | --- | --- | --- |
| Sex | Age | Population | Sample | Population | Sample | Population | Sample | Population | Sample | Population | Sample | Population | Sample | Population | Sample |
| Male | 20 - 29 | 3,513,630 | 351,363 | 3,462,092 | 346,209 | 3,438,604 | 343,860 | 3,455,018 | 345,502 | 3,492,794 | 349,279 | 3,526,733 | 352,673 | 3,556,649 | 355,665 |
|  | 30 - 39 | 4,193,211 | 419,321 | 4,143,117 | 414,312 | 4,085,613 | 408,561 | 4,000,925 | 400,093 | 3,917,274 | 391,727 | 3,850,487 | 385,049 | 3,778,265 | 377,827 |
|  | 40 - 49 | 4,435,055 | 443,506 | 4,437,927 | 443,793 | 4,452,694 | 445,269 | 4,474,485 | 447,449 | 4,459,325 | 445,933 | 4,418,590 | 441,859 | 4,376,817 | 437,682 |
|  | 50 - 59 | 3,592,137 | 359,214 | 3,776,653 | 377,665 | 3,909,235 | 390,924 | 4,020,937 | 402,094 | 4,097,255 | 409,726 | 4,155,589 | 415,559 | 4,198,174 | 419,817 |
|  | 60 - 69 | 1,983,828 | 198,383 | 2,025,529 | 202,553 | 2,094,829 | 209,483 | 2,194,269 | 219,427 | 2,342,576 | 234,258 | 2,502,779 | 250,278 | 2,648,477 | 264,848 |
|  | 70 - 79 | 1,096,507 | 109,651 | 1,183,270 | 118,327 | 1,258,609 | 125,861 | 1,305,432 | 130,543 | 1,337,821 | 133,782 | 1,369,560 | 136,956 | 1,426,917 | 142,692 |
|  | Sum | 18,814,368 | 1,881,438 | 19,028,588 | 1,902,859 | 19,239,584 | 1,923,958 | 19,451,066 | 1,945,108 | 19,647,045 | 1,964,705 | 19,823,738 | 1,982,374 | 19,985,299 | 1,998,531 |
| Female | 20 - 29 | 3,246,006 | 324,601 | 3,180,980 | 318,098 | 3,137,257 | 313,726 | 3,130,169 | 313,017 | 3,146,887 | 314,689 | 3,169,220 | 316,922 | 3,194,583 | 319,458 |
|  | 30 - 39 | 4,032,981 | 403,298 | 3,990,002 | 399,000 | 3,933,220 | 393,322 | 3,846,194 | 384,619 | 3,760,633 | 376,063 | 3,688,221 | 368,822 | 3,610,380 | 361,038 |
|  | 40 - 49 | 4,273,990 | 427,399 | 4,275,492 | 427,549 | 4,303,860 | 430,386 | 4,336,399 | 433,640 | 4,323,989 | 432,399 | 4,297,638 | 429,764 | 4,263,677 | 426,368 |
|  | 50 - 59 | 3,583,577 | 358,358 | 3,760,656 | 376,066 | 3,877,090 | 387,709 | 3,979,647 | 397,965 | 4,051,530 | 405,153 | 4,092,336 | 409,234 | 4,131,629 | 413,163 |
|  | 60 - 69 | 2,166,711 | 216,671 | 2,192,207 | 219,221 | 2,248,556 | 224,856 | 2,335,678 | 233,568 | 2,482,042 | 248,204 | 2,648,485 | 264,849 | 2,789,625 | 278,963 |
|  | 70 - 79 | 1,574,933 | 157,493 | 1,662,217 | 166,222 | 1,735,945 | 173,595 | 1,775,989 | 177,599 | 1,793,149 | 179,315 | 1,804,079 | 180,408 | 1,845,299 | 184,530 |
|  | Sum | 18,878,198 | 1,887,820 | 19,061,554 | 1,906,156 | 19,235,928 | 1,923,594 | 19,404,076 | 1,940,408 | 19,558,230 | 1,955,823 | 19,699,979 | 1,969,999 | 19,835,193 | 1,983,520 |
| Total | | 37,692,566 | 3,769,258 | 38,090,142 | 3,809,015 | 38,475,512 | 3,847,552 | 38,855,142 | 3,885,516 | 39,205,275 | 3,920,528 | 39,523,717 | 3,952,373 | 39,820,492 | 3,982,051 |

**Supplementary Table 2.** Comparison of high-risk drinking between NHIS-HEALs and the National Health and Nutrition Examination Survey

|  | % | 2011 | 2012 | 2013 | 2014 | 2015 | 2016 | 2017 |
| --- | --- | --- | --- | --- | --- | --- | --- | --- |
| **NHIS-HEALs**  (health check-up cohort) | Male | 24.3 | 23.7 | 23.4 | 23.2 | 23.4 | 23.0 | 22.8 |
|  | Female | 5.1 | 5.0 | 5.3 | 5.3 | 5.7 | 5.7 | 5.9 |
|  | Total | 14.6 | 14.3 | 14.2 | 14.1 | 14.5 | 14.3 | 14.3 |
| **National Health and Nutrition Examination Survey** | Male | 23.2 | 21.9 | 19.7 | 20.7 | 20.8 | 21.2 | 21.0 |
|  | Female | 4.9 | 6.0 | 5.5 | 6.6 | 5.8 | 6.3 | 7.2 |
|  | Total | 14.1 | 13.9 | 12.6 | 13.5 | 13.3 | 13.8 | 14.2 |

**Supplementary Table 3.** Prevalence of alcoholic liver disease

|  | |  | | **Prevalence (%)** | | | | | | | | | | | | | |
| --- | --- | --- | --- | --- | --- | --- | --- | --- | --- | --- | --- | --- | --- | --- | --- | --- | --- |
| **Sex** | | **Age** | | **2011** | | **2012** | | **2013** | | **2014** | | **2015** | | **2016** | | **2017** | |
| **Social drinker** | | | | | | | | | | | | | | | | | |
| Male | | 20 – 29 | | 0.10 | | 0.12 | | 0.08 | | 0.06 | | 0.08 | | 0.07 | | 0.05 | |
|  |  | 30 – 39 | | 0.22 | | 0.20 | | 0.17 | | 0.13 | | 0.13 | | 0.13 | | 0.12 | |
|  |  | 40 – 49 | | 0.35 | | 0.33 | | 0.27 | | 0.25 | | 0.23 | | 0.26 | | 0.21 | |
|  |  | 50 – 59 | | 0.56 | | 0.50 | | 0.49 | | 0.41 | | 0.42 | | 0.38 | | 0.36 | |
|  |  | 60 – 69 | | 0.69 | | 0.65 | | 0.55 | | 0.45 | | 0.52 | | 0.50 | | 0.55 | |
|  |  | 70 – 79 | | 0.55 | | 0.62 | | 0.49 | | 0.43 | | 0.50 | | 0.46 | | 0.49 | |
|  |  | Sum | | 0.33 | | 0.31 | | 0.27 | | 0.23 | | 0.24 | | 0.24 | | 0.23 | |
| Female | | 20 – 29 | | 0.03 | | 0.03 | | 0.02 | | 0.01 | | 0.02 | | 0.01 | | 0.01 | |
|  |  | 30 – 39 | | 0.04 | | 0.04 | | 0.03 | | 0.03 | | 0.02 | | 0.03 | | 0.02 | |
|  |  | 40 – 49 | | 0.08 | | 0.08 | | 0.06 | | 0.07 | | 0.04 | | 0.06 | | 0.05 | |
|  |  | 50 – 59 | | 0.20 | | 0.14 | | 0.13 | | 0.13 | | 0.11 | | 0.11 | | 0.11 | |
|  |  | 60 – 69 | | 0.16 | | 0.16 | | 0.13 | | 0.12 | | 0.09 | | 0.13 | | 0.14 | |
|  |  | 70 – 79 | | 0.07 | | 0.13 | | 0.20 | | 0.08 | | 0.08 | | 0.16 | | 0.11 | |
|  |  | Sum | | 0.07 | | 0.07 | | 0.06 | | 0.05 | | 0.04 | | 0.05 | | 0.05 | |
| Total | | | | 0.21 | | 0.20 | | 0.17 | | 0.15 | | 0.15 | | 0.15 | | 0.15 | |
| **High risk drinker** | | | | | | | | | | | | | | | | |  |
| Male | 20 – 29 | | 0.32 | | 0.24 | | 0.21 | | 0.19 | | 0.20 | | 0.19 | | 0.19 | |  |
|  | 30 – 39 | | 0.59 | | 0.51 | | 0.47 | | 0.45 | | 0.48 | | 0.48 | | 0.46 | |  |
|  | 40 – 49 | | 1.19 | | 1.24 | | 1.10 | | 1.07 | | 1.01 | | 1.07 | | 1.03 | |  |
|  | 50 – 59 | | 2.08 | | 2.05 | | 1.96 | | 1.80 | | 1.73 | | 1.67 | | 1.60 | |  |
|  | 60 – 69 | | 2.74 | | 2.67 | | 2.49 | | 2.35 | | 2.42 | | 2.43 | | 2.37 | |  |
|  | 70 – 79 | | 2.72 | | 2.50 | | 2.46 | | 2.56 | | 2.67 | | 2.61 | | 2.35 | |  |
|  | Sum | | 1.20 | | 1.19 | | 1.13 | | 1.08 | | 1.08 | | 1.10 | | 1.07 | |  |
| Female | 20 – 29 | | 0.09 | | 0.06 | | 0.07 | | 0.08 | | 0.07 | | 0.05 | | 0.07 | |  |
|  | 30 – 39 | | 0.25 | | 0.23 | | 0.19 | | 0.17 | | 0.20 | | 0.17 | | 0.23 | |  |
|  | 40 – 49 | | 0.58 | | 0.70 | | 0.72 | | 0.66 | | 0.66 | | 0.71 | | 0.68 | |  |
|  | 50 – 59 | | 0.97 | | 1.01 | | 0.94 | | 0.98 | | 1.02 | | 0.85 | | 1.05 | |  |
|  | 60 – 69 | | 1.03 | | 0.92 | | 0.76 | | 1.12 | | 0.85 | | 0.82 | | 0.85 | |  |
|  | 70 – 79 | | 1.46 | | 1.00 | | 0.76 | | 0.84 | | 1.60 | | 1.61 | | 1.03 | |  |
|  | Sum | | 0.37 | | 0.39 | | 0.37 | | 0.38 | | 0.38 | | 0.36 | | 0.40 | |  |
| Total | | | 1.05 | | 1.05 | | 0.99 | | 0.95 | | 0.94 | | 0.95 | | 0.93 | |  |

**Supplementary Table 4.** Prevalence of liver cirrhosis

|  | |  | **Prevalence (%)** | | | | | | |
| --- | --- | --- | --- | --- | --- | --- | --- | --- | --- |
| **Sex** | | **Age** | **2011** | **2012** | **2013** | **2014** | **2015** | **2016** | **2017** |
| **Social drinker** | | | | | | | | | |
| Male | | 20 – 29 | 0.01 | 0.01 | 0.01 | 0.01 | 0.00 | 0.01 | 0.01 |
|  |  | 30 – 39 | 0.04 | 0.02 | 0.03 | 0.04 | 0.04 | 0.03 | 0.04 |
|  |  | 40 – 49 | 0.16 | 0.15 | 0.15 | 0.16 | 0.16 | 0.15 | 0.13 |
|  |  | 50 – 59 | 0.34 | 0.35 | 0.34 | 0.41 | 0.37 | 0.35 | 0.33 |
|  |  | 60 – 69 | 0.38 | 0.40 | 0.44 | 0.36 | 0.37 | 0.45 | 0.41 |
|  |  | 70 – 79 | 0.27 | 0.33 | 0.37 | 0.38 | 0.35 | 0.45 | 0.41 |
|  |  | Sum | 0.14 | 0.15 | 0.15 | 0.17 | 0.16 | 0.17 | 0.16 |
| Female | | 20 – 29 | 0.00 | 0.00 | 0.00 | 0.00 | 0.00 | 0.01 | 0.01 |
|  |  | 30 – 39 | 0.01 | 0.01 | 0.01 | 0.01 | 0.01 | 0.01 | 0.01 |
|  |  | 40 – 49 | 0.04 | 0.05 | 0.03 | 0.04 | 0.04 | 0.04 | 0.04 |
|  |  | 50 – 59 | 0.10 | 0.10 | 0.11 | 0.12 | 0.11 | 0.12 | 0.08 |
|  |  | 60 – 69 | 0.16 | 0.15 | 0.15 | 0.16 | 0.16 | 0.18 | 0.20 |
|  |  | 70 – 79 | 0.17 | 0.13 | 0.13 | 0.15 | 0.22 | 0.14 | 0.26 |
|  |  | Sum | 0.03 | 0.04 | 0.04 | 0.04 | 0.04 | 0.05 | 0.04 |
| Total | | | 0.09 | 0.10 | 0.10 | 0.11 | 0.11 | 0.11 | 0.11 |
| **High risk drinker** | | | | | | | | | |
| Male | 20 – 29 | | 0.01 | 0.01 | 0.01 | 0.01 | 0.00 | 0.00 | 0.01 |
|  | 30 – 39 | | 0.02 | 0.02 | 0.02 | 0.02 | 0.03 | 0.02 | 0.02 |
|  | 40 – 49 | | 0.15 | 0.18 | 0.16 | 0.14 | 0.14 | 0.15 | 0.15 |
|  | 50 – 59 | | 0.40 | 0.48 | 0.44 | 0.41 | 0.41 | 0.40 | 0.37 |
|  | 60 – 69 | | 0.72 | 0.71 | 0.70 | 0.62 | 0.68 | 0.70 | 0.65 |
|  | 70 – 79 | | 0.92 | 0.96 | 0.80 | 1.03 | 0.64 | 0.93 | 0.92 |
|  | Sum | | 0.20 | 0.23 | 0.22 | 0.21 | 0.21 | 0.22 | 0.22 |
| Female | 20 – 29 | | 0.00 | 0.00 | 0.00 | 0.01 | 0.00 | 0.00 | 0.00 |
|  | 30 – 39 | | 0.01 | 0.02 | 0.02 | 0.05 | 0.02 | 0.01 | 0.02 |
|  | 40 – 49 | | 0.09 | 0.12 | 0.17 | 0.13 | 0.12 | 0.17 | 0.12 |
|  | 50 – 59 | | 0.13 | 0.27 | 0.26 | 0.32 | 0.22 | 0.26 | 0.33 |
|  | 60 – 69 | | 0.23 | 0.40 | 0.34 | 0.40 | 0.15 | 0.34 | 0.34 |
|  | 70 – 79 | | 0.42 | 0.20 | 0.19 | 0.17 | 0.16 | 0.32 | 0.29 |
|  | Sum | | 0.05 | 0.08 | 0.08 | 0.09 | 0.07 | 0.09 | 0.09 |
| Total | | | 0.17 | 0.20 | 0.19 | 0.19 | 0.18 | 0.20 | 0.19 |

**Supplementary Table 5.** Prevalence of hepatocellular carcinoma

|  | |  | | **Prevalence (%)** | | | | | | | | | | | |
| --- | --- | --- | --- | --- | --- | --- | --- | --- | --- | --- | --- | --- | --- | --- | --- |
| **Sex** | | **Age** | | **2011** | **2012** | | **2013** | | **2014** | | **2015** | | **2016** | **2017** | |
| **Social drinker** | | | | | | | | | | | | | | | |
| Male | | 20 – 29 | | 0.00 | 0.00 | | 0.00 | | 0.00 | | 0.00 | | 0.00 | 0.00 | |
|  |  | 30 – 39 | | 0.00 | 0.01 | | 0.00 | | 0.01 | | 0.01 | | 0.01 | 0.00 | |
|  |  | 40 – 49 | | 0.04 | 0.04 | | 0.02 | | 0.03 | | 0.03 | | 0.04 | 0.04 | |
|  |  | 50 – 59 | | 0.10 | 0.12 | | 0.11 | | 0.11 | | 0.14 | | 0.12 | 0.11 | |
|  |  | 60 – 69 | | 0.14 | 0.16 | | 0.19 | | 0.20 | | 0.16 | | 0.19 | 0.23 | |
|  |  | 70 – 79 | | 0.12 | 0.25 | | 0.15 | | 0.26 | | 0.16 | | 0.23 | 0.25 | |
|  |  | Sum | | 0.04 | 0.05 | | 0.04 | | 0.06 | | 0.06 | | 0.06 | 0.07 | |
| Female | | 20 – 29 | | 0.00 | 0.00 | | 0.00 | | 0.00 | | 0.00 | | 0.00 | 0.00 | |
|  |  | 30 – 39 | | 0.00 | 0.00 | | 0.00 | | 0.00 | | 0.00 | | 0.00 | 0.00 | |
|  |  | 40 – 49 | | 0.01 | 0.01 | | 0.01 | | 0.01 | | 0.01 | | 0.01 | 0.01 | |
|  |  | 50 – 59 | | 0.02 | 0.01 | | 0.02 | | 0.03 | | 0.03 | | 0.03 | 0.03 | |
|  |  | 60 – 69 | | 0.04 | 0.02 | | 0.04 | | 0.05 | | 0.04 | | 0.03 | 0.03 | |
|  |  | 70 – 79 | | 0.09 | 0.06 | | 0.04 | | 0.06 | | 0.08 | | 0.04 | 0.09 | |
|  |  | Sum | | 0.01 | 0.01 | | 0.01 | | 0.01 | | 0.01 | | 0.01 | 0.01 | |
| Total | | | | 0.03 | 0.03 | | 0.03 | | 0.03 | | 0.03 | | 0.04 | 0.04 | |
| **High risk drinker** | | | | | | | | | | | | | | |  |
| Male | 20 – 29 | | 0.00 | | 0.00 | 0.00 | | 0.00 | | 0.00 | | 0.00 | | 0.00 |  |
|  | 30 – 39 | | 0.00 | | 0.01 | 0.00 | | 0.00 | | 0.00 | | 0.00 | | 0.01 |  |
|  | 40 – 49 | | 0.02 | | 0.02 | 0.03 | | 0.02 | | 0.03 | | 0.03 | | 0.02 |  |
|  | 50 – 59 | | 0.10 | | 0.10 | 0.11 | | 0.07 | | 0.09 | | 0.08 | | 0.08 |  |
|  | 60 – 69 | | 0.17 | | 0.16 | 0.20 | | 0.20 | | 0.19 | | 0.20 | | 0.19 |  |
|  | 70 – 79 | | 0.24 | | 0.21 | 0.33 | | 0.32 | | 0.28 | | 0.36 | | 0.30 |  |
|  | Sum | | 0.04 | | 0.05 | 0.06 | | 0.05 | | 0.05 | | 0.06 | | 0.05 |  |
| Female | 20 – 29 | | 0.00 | | 0.00 | 0.00 | | 0.00 | | 0.00 | | 0.00 | | 0.00 |  |
|  | 30 – 39 | | 0.00 | | 0.00 | 0.00 | | 0.00 | | 0.00 | | 0.00 | | 0.00 |  |
|  | 40 – 49 | | 0.00 | | 0.01 | 0.01 | | 0.00 | | 0.01 | | 0.00 | | 0.00 |  |
|  | 50 – 59 | | 0.04 | | 0.02 | 0.02 | | 0.05 | | 0.04 | | 0.06 | | 0.05 |  |
|  | 60 – 69 | | 0.19 | | 0.08 | 0.00 | | 0.07 | | 0.06 | | 0.05 | | 0.05 |  |
|  | 70 – 79 | | 0.21 | | 0.00 | 0.00 | | 0.00 | | 0.00 | | 0.16 | | 0.15 |  |
|  | Sum | | 0.01 | | 0.01 | 0.01 | | 0.01 | | 0.01 | | 0.01 | | 0.01 |  |
| Total | | | 0.04 | | 0.04 | 0.05 | | 0.04 | | 0.04 | | 0.05 | | 0.04 |  |

**Supplementary Table 6.** 3-year mortality

|  |  | 2011  3-year follow-up  (2012-2014) | | | 2012  3-year follow-up  (2013-2015) | | | 2013  3-year follow-up  (2014-2016) | | | 2014  3-year follow-up  (2015-2017) | | | 2015  3-year follow-up  (2016-2018) | | | 2016  3-year follow-up  (2017-2019) | | | 2017  3-year follow-up  (2018-2020) | | |
| --- | --- | --- | --- | --- | --- | --- | --- | --- | --- | --- | --- | --- | --- | --- | --- | --- | --- | --- | --- | --- | --- | --- |
| Sex | Age | Cohort | Incidence  (n) | Incidence  rate (%) | Cohort | Incidence  (n) | Incidence  rate (%) | Cohort | Incidence  (n) | Incidence  rate (%) | Cohort | Incidence  (n) | Incidence  rate (%) | Cohort | Incidence  (n) | Incidence  rate (%) | Cohort | Incidence  (n) | Incidence  rate (%) | Cohort | Incidence  (n) | Incidence  rate (%) |
| **Social drinker** | | | | | | | | | | | | | | | | | | | | | | |
| Male | 20 – 29 | 72610 | 87 | 0.12 | 74378 | 74 | 0.10 | 76492 | 85 | 0.11 | 77237 | 79 | 0.10 | 75836 | 81 | 0.11 | 75221 | 72 | 0.10 | 74777 | 70 | 0.09 |
|  | 30 – 39 | 81979 | 117 | 0.14 | 84020 | 81 | 0.10 | 83540 | 95 | 0.11 | 84250 | 83 | 0.10 | 79588 | 89 | 0.11 | 76691 | 91 | 0.12 | 75951 | 69 | 0.09 |
|  | 40 – 49 | 71585 | 224 | 0.31 | 74268 | 234 | 0.32 | 74877 | 189 | 0.25 | 77993 | 182 | 0.23 | 76144 | 173 | 0.23 | 75135 | 178 | 0.24 | 75451 | 196 | 0.26 |
|  | 50 – 59 | 51378 | 349 | 0.68 | 56457 | 395 | 0.70 | 56843 | 385 | 0.68 | 61782 | 375 | 0.61 | 61630 | 334 | 0.54 | 61663 | 327 | 0.53 | 63744 | 340 | 0.53 |
|  | 60 – 69 | 24414 | 433 | 1.77 | 26051 | 446 | 1.71 | 26615 | 416 | 1.56 | 28979 | 449 | 1.55 | 31635 | 427 | 1.35 | 33669 | 426 | 1.27 | 36530 | 469 | 1.28 |
|  | Sum | 301966 | 1210 | 0.40 | 315174 | 1230 | 0.39 | 318367 | 1170 | 0.37 | 330241 | 1168 | 0.35 | 324833 | 1104 | 0.34 | 322379 | 1094 | 0.34 | 326453 | 1144 | 0.35 |
| Fe  male | 20 – 29 | 71020 | 29 | 0.04 | 71552 | 29 | 0.04 | 71674 | 29 | 0.04 | 74062 | 32 | 0.04 | 71950 | 30 | 0.04 | 71835 | 28 | 0.04 | 73380 | 29 | 0.04 |
|  | 30 – 39 | 80020 | 56 | 0.07 | 81336 | 48 | 0.06 | 81654 | 49 | 0.06 | 83570 | 47 | 0.06 | 82193 | 66 | 0.08 | 80331 | 37 | 0.05 | 81835 | 42 | 0.05 |
|  | 40 – 49 | 69289 | 87 | 0.13 | 70281 | 93 | 0.13 | 73174 | 76 | 0.10 | 77248 | 80 | 0.10 | 78023 | 88 | 0.11 | 77969 | 80 | 0.10 | 80804 | 69 | 0.09 |
|  | 50 – 59 | 33861 | 105 | 0.31 | 36777 | 95 | 0.26 | 39249 | 83 | 0.21 | 42843 | 92 | 0.21 | 45588 | 83 | 0.18 | 47031 | 98 | 0.21 | 50555 | 106 | 0.21 |
|  | 60 – 69 | 10476 | 65 | 0.62 | 10931 | 66 | 0.60 | 11346 | 62 | 0.55 | 12550 | 64 | 0.51 | 14074 | 60 | 0.43 | 15584 | 77 | 0.49 | 17465 | 89 | 0.51 |
|  | Sum | 264666 | 342 | 0.13 | 270877 | 331 | 0.12 | 277097 | 299 | 0.11 | 290273 | 315 | 0.11 | 291828 | 327 | 0.11 | 292750 | 320 | 0.11 | 304039 | 335 | 0.11 |
| Total | | c | 1552 | 0.27 | 586051 | 1561 | 0.27 | 595464 | 1469 | 0.25 | 620514 | 1483 | 0.24 | 616661 | 1431 | 0.23 | 615129 | 1414 | 0.23 | 630492 | 1479 | 0.23 |
| **High risk drinker** | | | | | | | | | | | | | | | | | | | | | | |
| Male | 20 – 29 | 88292 | 132 | 0.15 | 83677 | 135 | 0.16 | 80116 | 136 | 0.17 | 79156 | 117 | 0.15 | 80420 | 109 | 0.14 | 78307 | 93 | 0.12 | 78112 | 118 | 0.15 |
|  | 30 – 39 | 109604 | 208 | 0.19 | 105999 | 204 | 0.19 | 103386 | 193 | 0.19 | 100585 | 162 | 0.16 | 100064 | 155 | 0.15 | 96927 | 155 | 0.16 | 94946 | 175 | 0.18 |
|  | 40 – 49 | 131123 | 566 | 0.43 | 129136 | 612 | 0.47 | 128683 | 551 | 0.43 | 128185 | 513 | 0.40 | 129772 | 513 | 0.40 | 126875 | 506 | 0.40 | 124298 | 426 | 0.34 |
|  | 50 – 59 | 88020 | 1013 | 1.15 | 92556 | 962 | 1.04 | 97183 | 1036 | 1.07 | 100346 | 899 | 0.90 | 105648 | 931 | 0.88 | 107819 | 935 | 0.87 | 109781 | 928 | 0.85 |
|  | 60 – 69 | 32769 | 858 | 2.62 | 33471 | 833 | 2.49 | 34261 | 778 | 2.27 | 36497 | 813 | 2.23 | 39022 | 784 | 2.01 | 41696 | 809 | 1.94 | 44853 | 860 | 1.92 |
|  | Sum | 449808 | 2777 | 0.62 | 444839 | 2746 | 0.62 | 443629 | 2694 | 0.61 | 444769 | 2504 | 0.56 | 454926 | 2492 | 0.55 | 451624 | 2498 | 0.55 | 451990 | 2507 | 0.55 |
| Fe  male | 20 – 29 | 37311 | 32 | 0.09 | 37242 | 29 | 0.08 | 37583 | 25 | 0.07 | 39151 | 18 | 0.05 | 42458 | 31 | 0.07 | 42281 | 34 | 0.08 | 44128 | 37 | 0.08 |
|  | 30 – 39 | 27230 | 58 | 0.21 | 25213 | 52 | 0.21 | 28646 | 35 | 0.12 | 26550 | 41 | 0.15 | 29491 | 42 | 0.14 | 29812 | 36 | 0.12 | 30954 | 50 | 0.16 |
|  | 40 – 49 | 21206 | 61 | 0.29 | 21562 | 68 | 0.32 | 22821 | 74 | 0.32 | 23901 | 63 | 0.26 | 25806 | 64 | 0.25 | 26597 | 76 | 0.29 | 27396 | 90 | 0.33 |
|  | 50 – 59 | 11350 | 62 | 0.55 | 12023 | 57 | 0.47 | 12745 | 73 | 0.57 | 13011 | 65 | 0.50 | 14229 | 70 | 0.49 | 14527 | 54 | 0.37 | 15059 | 69 | 0.46 |
|  | 60 – 69 | 2133 | 22 | 1.03 | 2509 | 36 | 1.43 | 2621 | 20 | 0.76 | 3023 | 32 | 1.06 | 3401 | 32 | 0.94 | 3772 | 35 | 0.93 | 4351 | 40 | 0.92 |
|  | Sum | 99230 | 235 | 0.24 | 98549 | 242 | 0.25 | 104416 | 227 | 0.22 | 105636 | 219 | 0.21 | 115385 | 239 | 0.21 | 116989 | 235 | 0.20 | 121888 | 286 | 0.23 |
| Total | | 549038 | 3012 | 0.55 | 543388 | 2988 | 0.55 | 548045 | 2921 | 0.53 | 550405 | 2723 | 0.49 | 570311 | 2731 | 0.48 | 568613 | 2733 | 0.48 | 573878 | 2793 | 0.49 |

**Supplementary Table 7.** Comparison of alcoholic liver disease incidence rates in between NHIS-HEAL cohort and claims data

|  |  | 2012 | 2013 | 2014 | 2015 | 2016 | 2017 |
| --- | --- | --- | --- | --- | --- | --- | --- |
| **NHIS-HEALs**  (health check-up cohort) | Male | 0.69% | 0.64% | 0.60% | 0.60% | 0.59% | 0.57% |
|  | Female | 0.10% | 0.10% | 0.08% | 0.08% | 0.09% | 0.09% |
|  | Total | 0.39% | 0.37% | 0.34% | 0.34% | 0.34% | 0.33% |
| **Claim data** | Male | 0.69% | 0.65% | 0.59% | 0.59% | 0.58% | 0.57% |
|  | Female | 0.12% | 0.11% | 0.10% | 0.10% | 0.10% | 0.10% |
|  | Total | 0.41% | 0.38% | 0.35% | 0.35% | 0.34% | 0.33% |

**Supplementary Table 8.** Comparison of comorbidities between the alcoholic liver disease and the control group

|  | **2012** | | **2014** | | **2016** | | **2018** | | **2020** | | **2021** | |
| --- | --- | --- | --- | --- | --- | --- | --- | --- | --- | --- | --- | --- |
|  | ALD  (n=160727) | Control  (n=642908) | ALD  (n=144229) | Control  (n=576916) | ALD  (n=143205) | Control  (n=572820) | ALD  (n=139829) | Control  (n=559316) | ALD  (n=124403) | Control  (n=497612) | ALD  (n=119630) | Control  (n=478520) |
| Charlson Comorbidity Index | 2.67±1.89 | 0.94±1.44 | 2.75±1.94 | 0.99±1.48 | 2.82±1.97 | 1.07±1.55 | 2.91±2.03 | 1.16±1.6 | 2.88±2.06 | 1.12±1.62 | 2.87±2.08 | 1.14±1.63 |

|  |  | 2011 | | 2012 | | 2013 | | 2014 | | 2015 | | 2016 | | 2017 | |
| --- | --- | --- | --- | --- | --- | --- | --- | --- | --- | --- | --- | --- | --- | --- | --- |
| Sex | Age | Proportion (%) | SE (%) | Proportion (%) | SE (%) | Proportion (%) | SE (%) | Proportion (%) | SE (%) | Proportion (%) | SE (%) | Proportion (%) | SE (%) | Proportion (%) | SE (%) |
|  | | | | | | | | | | | | | | | |
| Male | 20 – 29 | 75.0 | 0.04 | 74.9 | 0.04 | 74.7 | 0.04 | 74.1 | 0.04 | 73.5 | 0.04 | 72.0 | 0.04 | 71.4 | 0.04 |
|  | 30 – 39 | 74.2 | 0.03 | 75.0 | 0.03 | 74.9 | 0.03 | 75.4 | 0.03 | 75.2 | 0.03 | 74.4 | 0.04 | 74.8 | 0.04 |
|  | 40 – 49 | 72.6 | 0.03 | 73.1 | 0.03 | 73.2 | 0.03 | 73.6 | 0.03 | 73.7 | 0.03 | 73.3 | 0.03 | 73.4 | 0.03 |
|  | 50 – 59 | 66.1 | 0.04 | 66.6 | 0.04 | 66.7 | 0.03 | 67.5 | 0.03 | 68.0 | 0.03 | 67.8 | 0.03 | 68.0 | 0.03 |
|  | 60 – 69 | 55.2 | 0.05 | 55.4 | 0.05 | 55.1 | 0.05 | 56.0 | 0.05 | 56.6 | 0.05 | 56.5 | 0.04 | 56.7 | 0.04 |
|  | 70 – 79 | 42.3 | 0.07 | 42.3 | 0.06 | 41.9 | 0.06 | 41.6 | 0.06 | 41.6 | 0.06 | 41.5 | 0.06 | 41.6 | 0.06 |
|  | Sum | 68.6 | 0.02 | 68.7 | 0.02 | 68.5 | 0.02 | 68.7 | 0.02 | 68.6 | 0.02 | 67.8 | 0.02 | 67.7 | 0.02 |
| Female | 20 – 29 | 52.4 | 0.04 | 53.7 | 0.04 | 54.8 | 0.04 | 56.8 | 0.04 | 58.5 | 0.04 | 58.3 | 0.04 | 59.9 | 0.04 |
|  | 30 – 39 | 40.2 | 0.03 | 40.0 | 0.03 | 42.7 | 0.03 | 43.3 | 0.04 | 45.7 | 0.04 | 46.6 | 0.04 | 49.3 | 0.04 |
|  | 40 – 49 | 32.0 | 0.03 | 32.4 | 0.03 | 33.9 | 0.03 | 35.3 | 0.03 | 36.6 | 0.03 | 37.3 | 0.03 | 39.2 | 0.03 |
|  | 50 – 59 | 19.9 | 0.04 | 20.2 | 0.04 | 21.0 | 0.04 | 21.7 | 0.03 | 22.9 | 0.03 | 23.4 | 0.03 | 24.6 | 0.03 |
|  | 60 – 69 | 9.3 | 0.05 | 9.7 | 0.05 | 9.8 | 0.05 | 10.6 | 0.05 | 11.2 | 0.04 | 11.8 | 0.04 | 12.5 | 0.04 |
|  | 70 – 79 | 5.3 | 0.05 | 5.2 | 0.05 | 4.9 | 0.05 | 4.9 | 0.05 | 5.1 | 0.05 | 5.1 | 0.05 | 5.3 | 0.05 |
|  | Sum | 30.1 | 0.02 | 30.2 | 0.02 | 31.1 | 0.02 | 31.8 | 0.02 | 32.9 | 0.02 | 33.2 | 0.02 | 34.4 | 0.02 |
| Total | | 49.3 | 0.01 | 49.4 | 0.01 | 49.8 | 0.01 | 50.3 | 0.01 | 50.8 | 0.01 | 50.6 | 0.01 | 51.1 | 0.01 |

**Supplementary Table 9.** Drinking rate more than once a week

**Supplementary Table 10.** population coverage, treatment coverage, and copayment in patients with alcohol use disorder and treatment

|  | **2011** | **2012** | **2013** | **2014** | **2015** | **2016** | **2017** |
| --- | --- | --- | --- | --- | --- | --- | --- |
| Number of patients | 149,041 | 157,383 | 157,793 | 160,980 | 166,842 | 168,982 | 175,811 |
| Average individual co-payment (won) | 227,613 | 227,767 | 231,624 | 239,225 | 241,568 | 236,447 | 225,118 |
| Insurer's cost per individual (won) | 1,342,049 | 1,293,445 | 1,318,693 | 1,376,505 | 1,396,688 | 1,354,258 | 1,261,405 |
| Total amount of individual co-payment (won) | 33,923,724,070 | 35,846,630,980 | 36,548,647,750 | 38,510,453,390 | 40,303,705,680 | 39,955,285,330 | 39,578,228,500 |
| Total amount of insurer's payment (won) | 200,020,393,220 | 203,566,306,040 | 208,080,564,520 | 221,589,806,870 | 233,026,214,820 | 228,845,170,500 | 221,768,829,280 |
| Total health insurance expenditure (won) | 233,944,117,290 | 239,412,937,020 | 244,629,212,270 | 260,100,260,260 | 273,329,920,500 | 268,800,455,830 | 261,347,057,780 |

**Supplementary Fig. 1** Proportions of social drinker and high risk drinker (%)

**
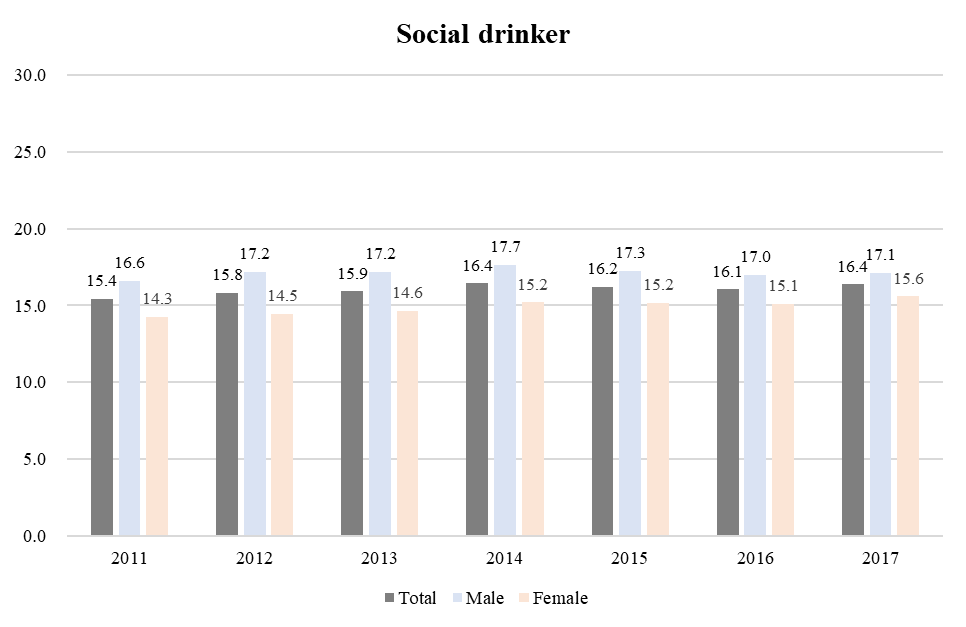
**

**
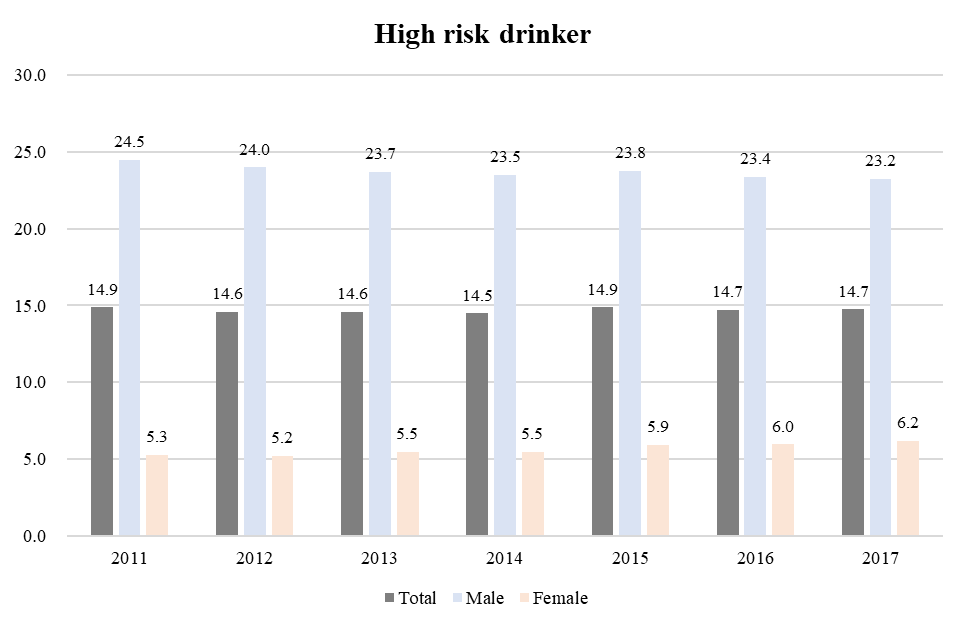
**

**Supplementary Fig. 2** Risk ratio of (A) alcoholic liver disease, (B) liver cirrhosis, and (C) hepatocellular carcinoma compared with social drinkers

**(A)**

**
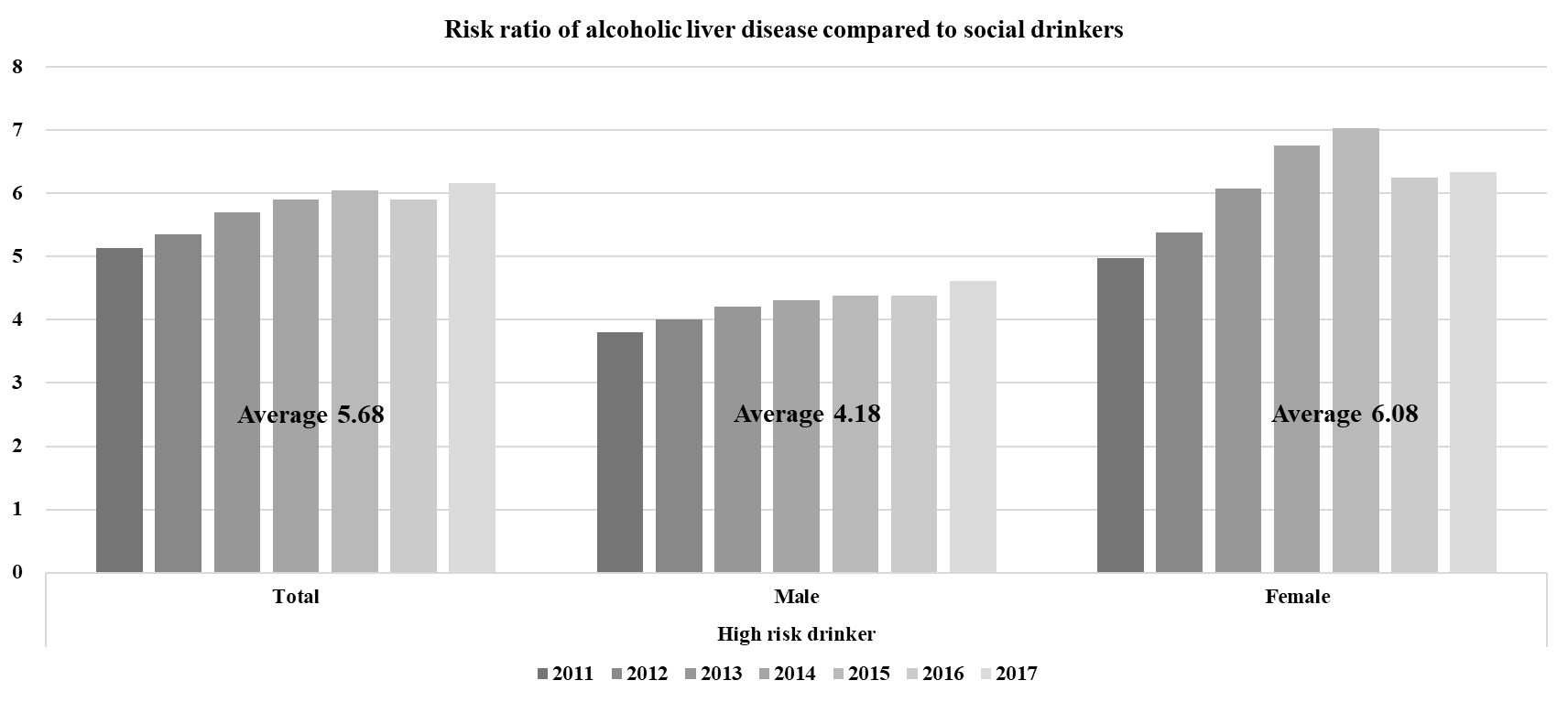
**

**(B)**

**
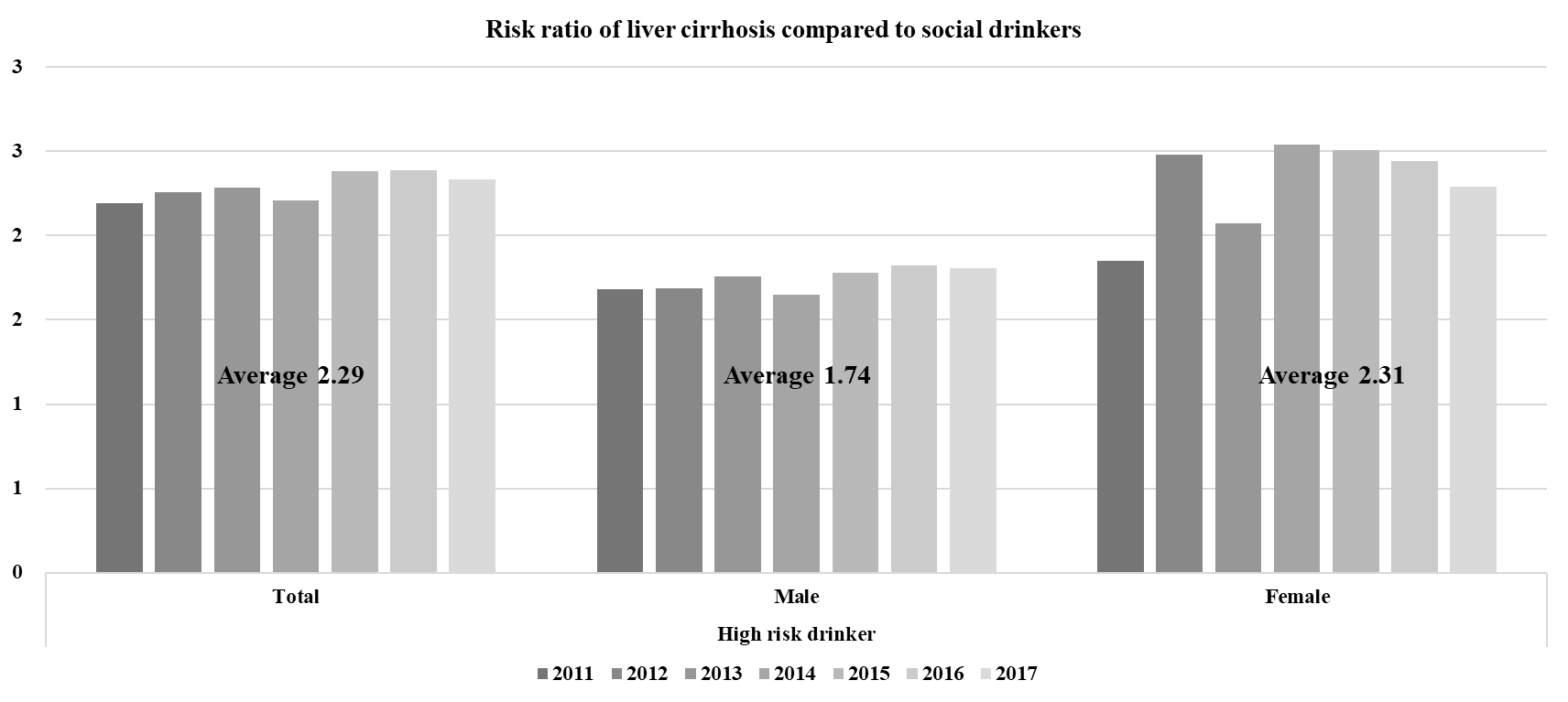
**

**(C)**

**
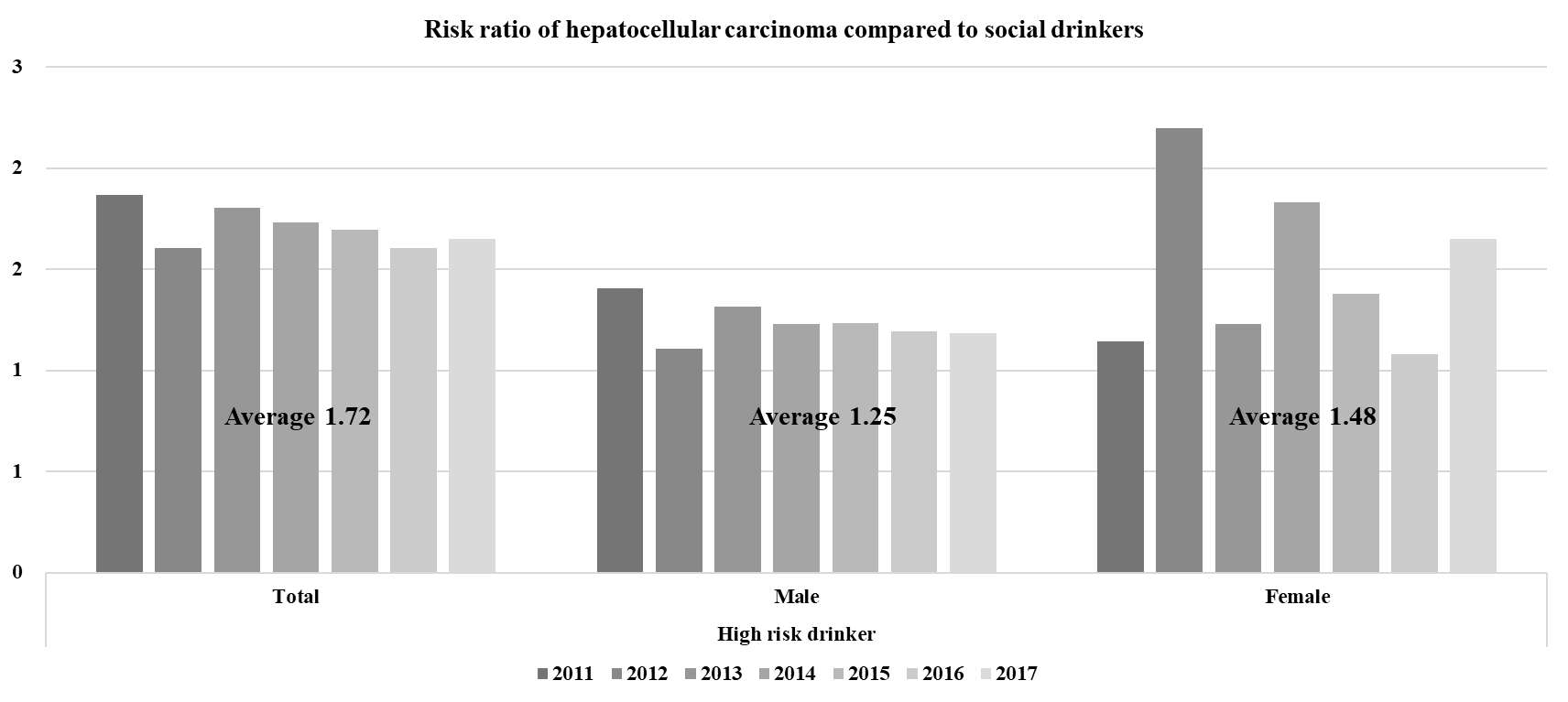
**

**Supplementary Fig. 3** Economic burden (2A) and healthcare utilization (2B) of alcoholic liver disease

**(A)**

**
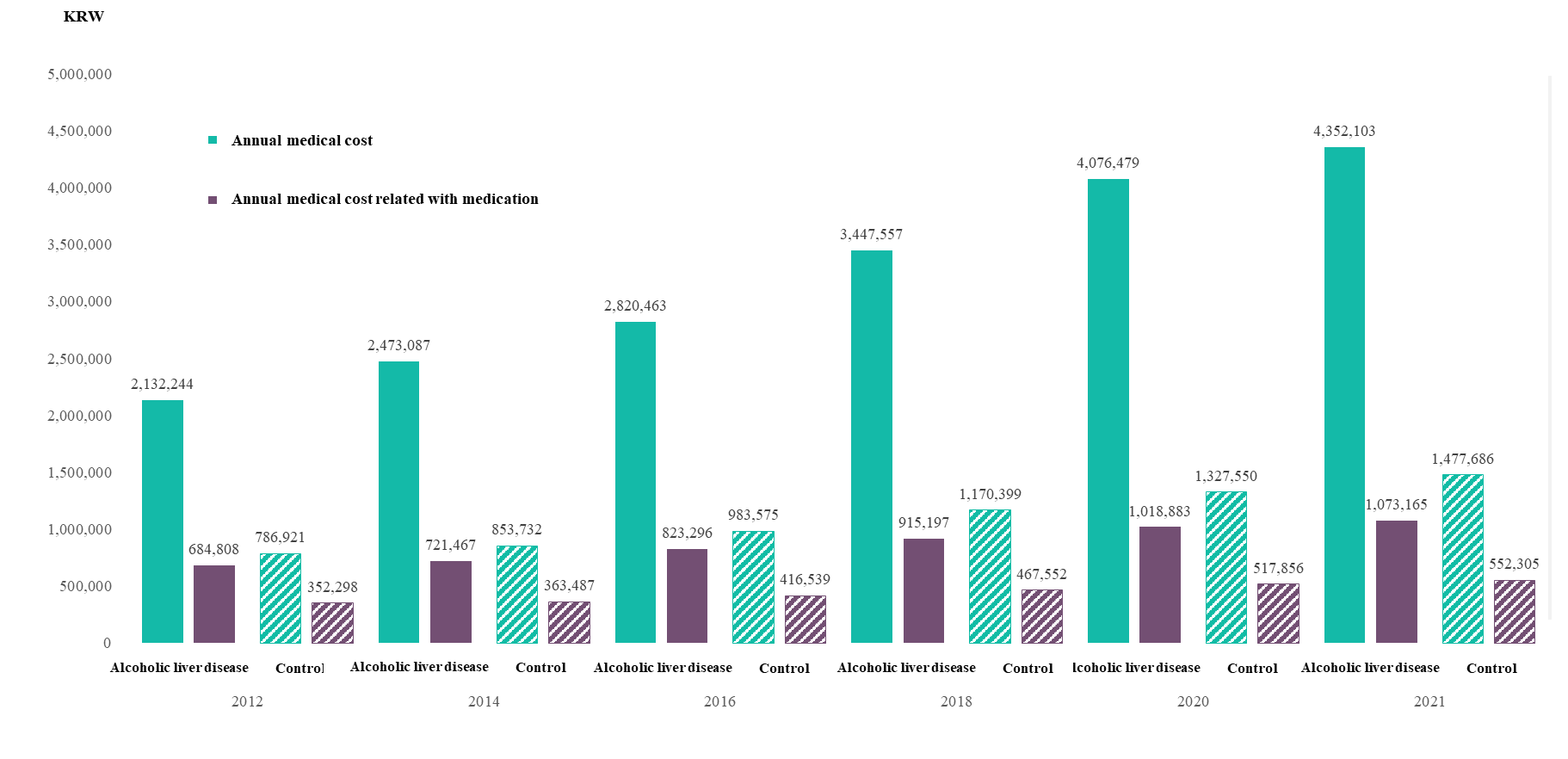
**

**(B)**

**
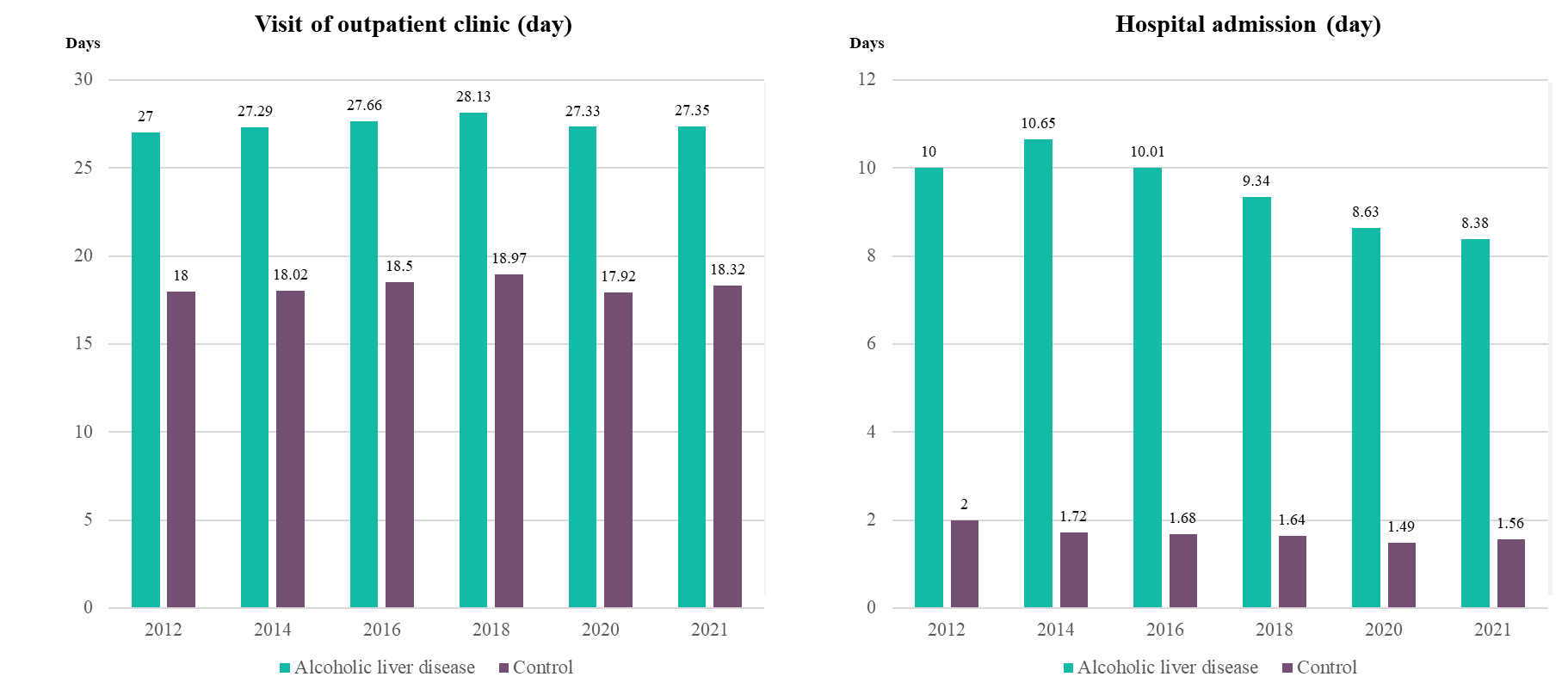
**

**Supplementary Fig. 4** Diagnosis of alcohol use disorder in patients with alcoholic liver disease (%)


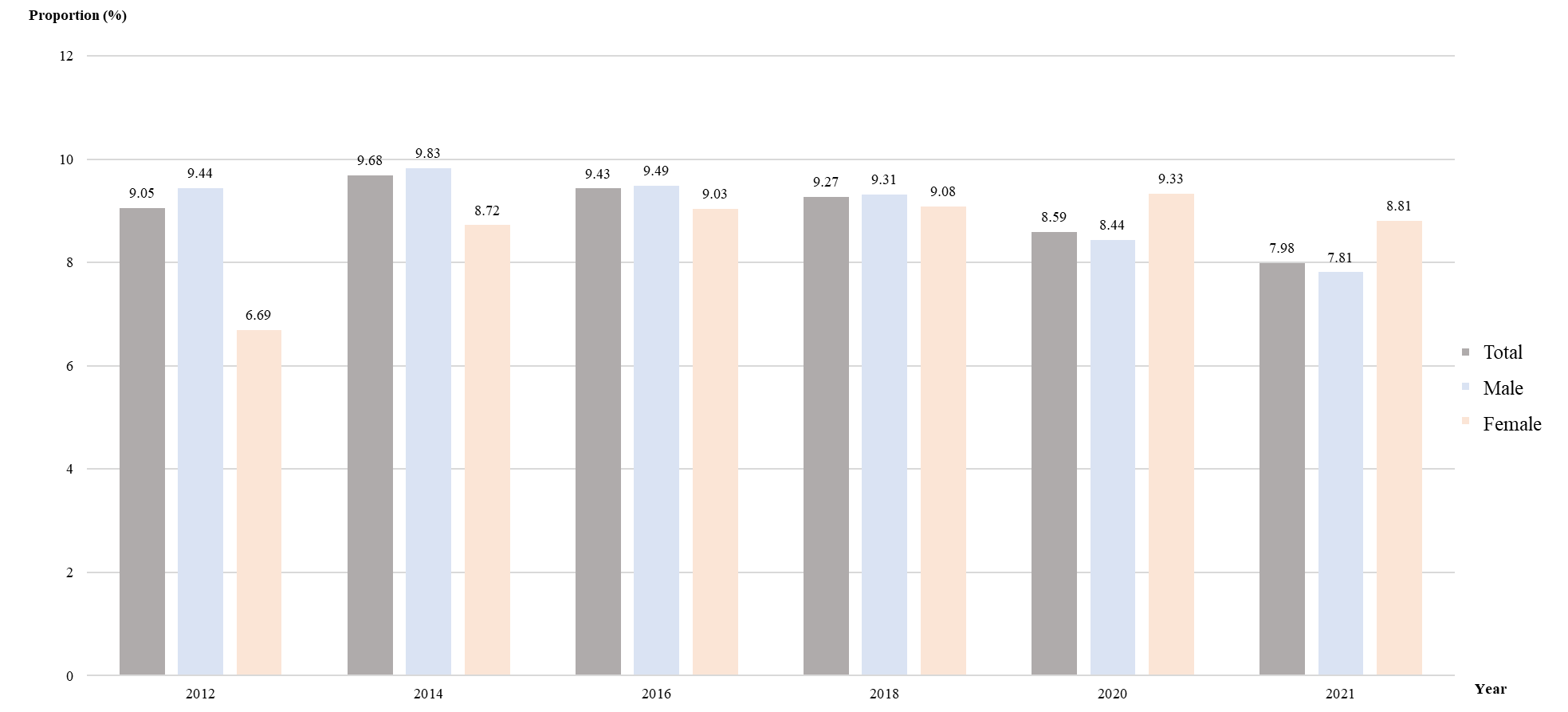

Supplement: Supplementary file 1 — Supplementary Material 1. [file 12889_2024_19321_MOESM1_ESM.docx]
